# GAL08, an Uncultivated Group of *Acidobacteria*, Is a Dominant Bacterial Clade in a Neutral Hot Spring

**DOI:** 10.3389/fmicb.2021.787651

**Published:** 2022-01-11

**Authors:** Ilona A. Ruhl, Andriy Sheremet, Chantel C. Furgason, Susanne Krause, Robert M. Bowers, Jessica K. Jarett, Triet M. Tran, Stephen E. Grasby, Tanja Woyke, Peter F. Dunfield

**Affiliations:** ^1^Department of Biological Sciences, University of Calgary, Calgary, AB, Canada; ^2^U.S. Department of Energy Joint Genome Institute, Berkeley, CA, United States; ^3^Department of Geoscience, University of Calgary, Calgary, AB, Canada; ^4^Geological Survey of Canada, Calgary, AB, Canada

**Keywords:** *Acidobacteria*, *Acidobacteriota*, uncultivated bacterium, GAL08, hot spring, thermophile, single-cell genomics

## Abstract

GAL08 are bacteria belonging to an uncultivated phylogenetic cluster within the phylum *Acidobacteria*. We detected a natural population of the GAL08 clade in sediment from a pH-neutral hot spring located in British Columbia, Canada. To shed light on the abundance and genomic potential of this clade, we collected and analyzed hot spring sediment samples over a temperature range of 24.2–79.8°C. Illumina sequencing of 16S rRNA gene amplicons and qPCR using a primer set developed specifically to detect the GAL08 16S rRNA gene revealed that absolute and relative abundances of GAL08 peaked at 65°C along three temperature gradients. Analysis of sediment collected over multiple years and locations revealed that the GAL08 group was consistently a dominant clade, comprising up to 29.2% of the microbial community based on relative read abundance and up to 4.7 × 10^5^ 16S rRNA gene copy numbers per gram of sediment based on qPCR. Using a medium quality threshold, 25 single amplified genomes (SAGs) representing these bacteria were generated from samples taken at 65 and 77°C, and seven metagenome-assembled genomes (MAGs) were reconstructed from samples collected at 45–77°C. Based on average nucleotide identity (ANI), these SAGs and MAGs represented three separate species, with an estimated average genome size of 3.17 Mb and GC content of 62.8%. Phylogenetic trees constructed from 16S rRNA gene sequences and a set of 56 concatenated phylogenetic marker genes both placed the three GAL08 bacteria as a distinct subgroup of the phylum *Acidobacteria*, representing a candidate order (*Ca.* Frugalibacteriales) within the class *Blastocatellia.* Metabolic reconstructions from genome data predicted a heterotrophic metabolism, with potential capability for aerobic respiration, as well as incomplete denitrification and fermentation. In laboratory cultivation efforts, GAL08 counts based on qPCR declined rapidly under atmospheric levels of oxygen but increased slightly at 1% (v/v) O_2_, suggesting a microaerophilic lifestyle.

## Introduction

The phylum *Acidobacteria* is one of the most abundant and phylogenetically diverse bacterial phyla in nature (Hugenholtz et al., 1998). The phylum has informally been divided into 26 subgroups based on 16S rRNA gene phylogeny, with subgroups 1, 3, 4, and 6 being the most abundant globally (Barns et al., 1999). The division of the phylum into subgroups was originally proposed by Hugenholtz et al. (1998), who considered a subgroup to be any group of two or more 16S rRNA sequences that were reproducibly monophyletic and unaffiliated with all other sequences within the phylum. The 26 subgroups do not correspond to any particular taxonomic rank but instead correspond to different class, order, or family rank taxa in release 132 of the Silva rRNA gene database (Pruesse et al., 2012). The Silva database lists 21 classes within *Acidobacteria*, while the GTDB genome-based database lists 14 classes and proposes the alternative phylum name *Acidobacteriota* (Oren et al., 2015; Parks et al., 2018). In either system, there are only 5 classes with validly published names: *Acidobacteria*, *Blastocatellia*, *Holophagae*, *Vicinamibacteria*, and *Thermoanaerobaculia* (Dedysh and Yilmaz, 2018; Parte, 2018).

Despite their ubiquity in environmental surveys, only 61 species of *Acidobacteria* have been validly published as of July 2021 according to the List of Prokaryotic Names with Standing in Nomenclature website (Parte et al., 2020). All cultivated species of *Acidobacteria* are chemoorganoheterotrophic, except for *Chloracidobacterium thermophilum*, a photoheterotroph (Tank and Bryant, 2015). Most of the cultivated *Acidobacteria* are aerobic, although some species can grow in different oxygen concentrations, and some facultative aerobes, microaerophiles, and obligate anaerobes have been described (Kielak et al., 2016). Different *Acidobacteria* can utilize both organic and inorganic nitrogen sources and can metabolize a variety of different carbohydrates, from simple sugars such as arabinose, fructose, glucose, mannose, and xylose to disaccharides such as lactose, maltose, cellobiose, sucrose, trehalose, and melibiose and polysaccharides such as xylan, starch, laminarin, chitin, and many others (Eichorst et al., 2018). Genomic analyses of *Acidobacteria* isolate genomes show adaptations for survival in environments with fluctuating nutrient availability (Eichorst et al., 2018). Despite the low number of cultured species, *Acidobacteria* are common in many environments, and therefore a large number of additional *Acidobacteria* genomes have been recovered as single amplified genomes (SAGs) and metagenome-assembled genomes (MAGs). At the time of publication, nearly 700 *Acidobacteria* genomes are available in the GTDB (Parks et al., 2018).

*Acidobacteria* have been detected in a wide range of habitats (Barns et al., 1999), including thermal environments. Environmental surveys have detected acidobacterial 16S rRNA gene sequences in thermally disturbed soils in Yellowstone National Park (Norris et al., 2002), hydrothermal sediments from a deep-sea vent (López-García et al., 2003), terrestrial fumaroles and mud pools (Glamoclija et al., 2004), and various hot springs (Stott et al., 2008). Only four acidobacterial isolates are thermophilic (Kielak et al., 2016): *Pyrinomonas methylaliphatogenes* and *Chloracidobacterium thermophilum* (class *Blastocatellia*), *Thermotomaculum hydrothermale* (class *Holophagae*), and *Thermoanaerobaculum aquaticum* (class *Thermoanaerobaculia*). With the only exception of *C. thermophilum*, all thermophilic isolates are heterotrophs. The two *Blastocatellia* isolates are microaerophilic (*C. thermophilum*) or aerobic (*P. methylaliphatogenes*), while *T. hydrothermale* and *T. aquaticum* are both strictly anaerobic.

While performing a large-scale survey of microbial communities in hot springs in Canada, we detected a high abundance of the uncultivated group “GAL08” (Ackerman, 2006) at the Dewar Creek hot spring in British Columbia (Sharp et al., 2014). Dewar Creek is a silicate-hosted neutral spring (pH 7.1–7.9) composed of meteoric water that migrates from a depth of up to 5 km of crustal rock (Grasby et al., 1999). It is one of the hottest geothermal springs in Canada, with a temperature of 80–86°C at the source (Grasby and Hutcheon, 2001). The high natural abundance of GAL08 in the Dewar Creek hot spring facilitated the construction of GAL08 SAGs and MAGs. In this study, we present ecological and genomic insights into these bacteria.

## Materials and Methods

### Sample Collection

The Dewar Creek hot spring is located at 49°55′ N and 116°28′ W in the Purcell Wilderness Conservancy of British Columbia, Canada. The water does not pool at the source but instead flows in three shallow channels (around 3 cm deep) over a tufa mound into an alpine creek (also named Dewar Creek). The temperature was measured *in situ*, and 40 mL samples of sediment were removed from below the flowing hot water. Collected sediment had sand-sized carbonate grains, was dark brown, and sometimes had a thin (less than 0.5 cm) top layer of brown-orange color. The samples were collected within several meters of grassy plant communities (Supplementary Figure 1). Samples were stored at ambient temperature for less than 24 h during transport from the field to the laboratory. Subsequently, samples were stored at −80°C [with 5% dimethylsulfoxide (DMSO) as a cryoprotectant] for preservation, −20°C for DNA extraction, and 4°C for cultivation. Six sampling trips were conducted over 8 years (2010–2017). Samples used for single-cell sorting were collected on September 15, 2010, and October 29, 2012; samples used in Illumina amplicon library analyses were collected on October 29, 2012, November 1, 2014, and August 24, 2015; samples tested for the abundance of GAL08 *via* qPCR with GAL08-specific primers were collected on November 6, 2011, August 24, 2015, and July 22, 2017. The 2017 samples were also used for enrichment attempts, as described below. On August 24, 2015, three sample transects were collected, one in each of three streams that flow out from a single source in a T-intersection pattern. Each transect began near the source and extended approximately 8 m downstream (covering a temperature range of 22.5–79.8°C).

### DNA Extraction

Frozen sediment samples were thawed and homogenized using the Precellys 24 Bead Mill Homogenizer (Bertin Instruments, Montigny-le-Bretonneux, France). DNA extraction was performed using the FastDNA Extraction Kit for Soil (MP Biomedicals, Santa Ana, CA, United States) with the following modifications: an additional purification step using 5.5 M guanidine thiocyanate was performed (Knief et al., 2003), and the extracted DNA was eluted in Qiagen Elution Buffer (QIAGEN, Toronto, ON, Canada). Quantification of DNA extracts was performed using the Qubit HS kit (Invitrogen, Carlsbad, CA, United States). Extract aliquots that were used for Illumina 16S rRNA gene sequencing were diluted to 5 ng μL^–1^ in preparation for amplification, as recommended by Illumina.

### PCR Amplification of 16S rRNA Genes

A sequence cluster belonging to the GAL08 group (henceforth called DChs_GAL08) was detected in preliminary surveys of the Dewar Creek hot spring in 2010 and 2011 (Sharp et al., 2014). In brief, samples were amplified using the universal primers 907fw (5′-AAACTYAAAKGAATTGRCGG-3′) and 1392rv (5′-ACGGGCGGTGTGTRC-3′), barcoded with a 10-nucleotide barcode, and sequenced using the 454 pyrosequencing platform (Sharp et al., 2014). Samples used in the current study were collected between 2012 and 2015 (32 samples in total), amplified using the *Bacteria*-specific primers 341fw (5′-CCTACGGGNGGCWGCAG-3′) and 785rv (5′-GACTACHVGGGTATCTAATCC-3′) (Herlemann et al., 2011), and processed for Illumina sequencing as follows. Amplicon libraries were prepared as described in the library preparation protocol of Illumina (Illumina Inc., San Diego, CA, United States): “16S Metagenomic Sequencing Library Preparation” (Part # 15044223 Rev. B), with the exception that Taq polymerase (Sigma-Aldrich, St. Louis, MO, United States), was used for the second PCR reaction. Libraries were quantified using the Qubit HS kit (Invitrogen) as per the instructions of the manufacturer, and the molarity was calculated using the formula provided in the library preparation protocol of Illumina. Following quantification, libraries were diluted to 4 nM, pooled, and prepared for sequencing on the MiSeq instrument as per the “Preparing Libraries for Sequencing on the MiSeq” protocol of Illumina (Part # 15039740 Rev. D). Libraries were sequenced using the MiSeq Reagent Kit version 3, 600 cycles (Illumina part number MS-102-3003).

### Data Analysis of 16S rRNA Gene Libraries

16S rRNA gene sequencing data were first processed using the Quantitative Insights into Microbial Ecology (QIIME) pipeline version 1.9.1 (Caporaso et al., 2010). In short, raw sequence data were demultiplexed, and the barcode sequences were removed on the MiSeq instrument. Forward and reverse reads were paired using a minimum overlap of 20 base pairs. Reads were then subjected to a quality control filter that removed all reads with a Phred quality score below 20. The reads remaining after quality control filtering were clustered into operational taxonomic units (OTUs) at 97% similarity and taxonomically classified using BLAST^1^ using the Silva 119 database (Quast et al., 2013). This analysis produced a single OTU belonging to DChs_GAL08 and was used to assess its relative abundance in the community. For the identification of all amplicon sequence variants (ASVs) of the DChs_GAL08 group, sequencing data were processed using the Quantitative Insights into Microbial Ecology 2 (QIIME2) pipeline version 2021.4 (Estaki et al., 2020). In short, raw sequence data were demultiplexed, and the barcode sequences were removed on the MiSeq instrument. Reads were denoised using DADA2 (Callahan et al., 2016) with no trim length. The reads remaining after quality control were taxonomically classified using the SILVA 138 SSU database (Quast et al., 2013).

### Phylogenetic Reconstruction

A 16S rRNA gene-based Bayesian tree was constructed to show the placement of DChs_GAL08 relative to other acidobacterial sequences from the Silva rRNA gene database release 138 (Quast et al., 2013). Full-length 16S rRNA sequences were extracted from the SAG data (see below) and aligned using MAFFT version 1.3.7 (Katoh and Standley, 2013) using the G-INS-I method. Sites containing more than 95% gaps were masked. The tree was constructed using MrBayes version 3.2.6 (Ronquist et al., 2012). Posterior probabilities were estimated using a Markov Chain Monte Carlo of 2 × 10^6^ interactions with a burn-in of 1 × 10^5^. Data were analyzed using a 4 × 4 nucleotide substitution model with a general time reversible (GTR) structure. Rate variation was set to gamma-distributed with a proportion of invariable sites. The database entries used in building the tree and the criteria for selecting these sequences can be found in Supplementary Table 1. All selected sequences were 1,395 bp or longer. In total, 153 sequences were used in the construction of the tree.

A maximum-likelihood multiple concatenated marker gene tree was built using selected DChs_GAL08 SAGs and MAGs (see below), along with 68 *Acidobacteria* genomes and 214 reference genomes belonging to six other phyla. Six genomes from the *Parcubacteria* were used as an outgroup. The 214 genomes were selected from a reduced set of bacterial isolate genomes available in the Integrated Microbial Genomes (IMG) database (Chen et al., 2021). The total set of (61,619) available genomes was first reduced by clustering the RNA polymerase beta-subunit gene at 65%; then, the reduced set was further minimized by screening for genomes belonging to phyla to which the DChs_GAL08 16S rRNA gene sequence shows the closest 16S rRNA gene identity (*Thermotogae*, *Aquificae*, *Thermodesulfobacteria*, *Firmicutes*, *Actinobacteria*, and *Bacteroidetes*, at 82–84%). The final set served as a reference database for downstream tree inference. In brief, to construct the tree, proteins were called using Prodigal version 2.6.3 (Hyatt et al., 2010), phylogenetic markers were extracted from the resulting output files using Hidden Markov Models of each of the 56 markers using HMMER version 3.1b2^2^, aligned using MAFFT version 7.221 (Katoh and Standley, 2013), and concatenated using an internal python script. The phylogenetic tree was inferred using IQ tree (Nguyen et al., 2015), using the reference database described above, to produce a maximum likelihood tree using 1,000 bootstraps. Visualization was produced in R using ape and ggtree (Yu et al., 2017) packages.

### Single Amplified Genome and Metagenome-Assembled Genome Generation and Annotation

Two sediment samples were collected at the Dewar Creek hot spring in 2010 and 2012 at 77 and 65°C, respectively. After cryopreservation using DMSO, samples were thawed and spun for 15 s to separate the sediment particles from the source-water supernatant. Even after up to 4 years of storage, intact cells were observed in the supernatant under bright-field microscopy. This material was used for single-cell sorting and SAG creation as described by Eloe-Fadrosh et al. (2016). In summary, single cells were isolated using fluorescence-activated cell sorting (FACS), and cellular DNA was released with alkaline cell lysis and subjected to whole-genome amplification (WGA) and sequencing. The SAG sequences were then assembled using SPAdes (Bankevich et al., 2012) and quality screened using ProDeGe (Tennessen et al., 2016).

Using this method, a total of 25 SAGs identified as GAL08 were obtained. SAGs ranged in estimated completeness from 97.2% to 14.2%, as estimated using CheckM (Parks et al., 2015). Four metagenomes from samples of the Dewar Creek site at 45, 65, 66, and 77°C (IMG IDs: 3300025105, 3300025094, 3300025775, and 3300006767) have also been previously assembled and binned into MAGs as described by Nayfach et al. (2021). Seven MAGs in these samples were identified as belonging to GAL08. All SAGs and MAGs with >30% estimated completeness are listed in Table 1.

**TABLE 1:** Single amplified genomes (SAGs) and metagenome-assembled genomes (MAGs) identified as GAL08 recovered in this study.

Species^a^	Type	Assembly size (Mbp)	Est. completeness %^b^	Est. Size (Mbp)^b^	Quality^b^	Est. contamination (%)^b^	IMG Taxon ID
Species 1	MAG	3.08	95.3	3.23	High	2.56	3300025105_19
	MAG	3.09	95.3	3.24	High	3.42	3300025775_16
	MAG	3.05	93.3	3.28	High	2.56	3300006767
	SAG	3.04	92.7	3.28	High	2.56	3300013894
	SAG	2.75	89.3	3.08	Medium	1.71	3300014485
	MAG	2.90	89.3	3.24	Medium	1.71	3300025094_20
	SAG	2.39	74.6	3.20	Medium	2.56	2616644824
	SAG	1.93	63.4	2.92	Medium	0.85	3300014036
	SAG	1.39	44.0	3.15	Low	0	3300022998
	SAG	1.23	36.5	3.36	Low	0	2634166204
	SAG	1.03	33.7	3.04	Low	0	3300010608
	7 more SAGs^a^		<30%				
Species 2	MAG	2.50	69.7	3.08	Medium	5.87	3300006767
	SAG	2.50	85.3	2.94	Medium	2.56	2706794759
	SAG	2.47	75.9	3.25	Medium	2.75	3300014484
	SAG	2.23	70.6	3.16	Medium	2.56	3300014483
	SAG	0.78	30.2	2.60	Low	0.85	3300014433
Species 3	MAG	3.28	96.6	3.39	High	2.56	3300025105_14
	SAG	2.14	63.6	3.35	Medium	0.85	2706794767
	SAG	1.80	62.5	3.15	Medium	0.85	3300014343
	MAG	2.01	56.4	3.57	Medium	0.85	3300025094_36
	SAG	1.38	47.5	2.89	Low	0	3300014464
	SAG	1.87	46.3	4.03	Low	0	2619618948
	SAG	1.18	34.3	3.43	Low	0	3300010653
	2 more SAGs*^b^*		<30%				

*Grouping of SAGs into species was made based on average nucleotide identity (ANI) comparisons (Figure 3). Completeness and contamination were estimated using CheckM (Parks et al., 2015). Quality was assessed as described by Bowers et al. (2017).*

*^a^IMG Taxon IDs: 3300013081, 3300010584, 3300010538, 3300010583, 3300014406, 2619618940, and 3300022972.*

*^b^IMG Taxon IDs: 3300014405 and 3300014434.*

Single amplified genomes and MAGs were annotated and analyzed in detail using the IMG platform of JGI (Chen et al., 2021). FeGenie was used to identify potential genes encoding iron metabolism using hidden Markov models (Garber et al., 2020). ANI comparisons of SAGs and MAGs were calculated using pyani^3^ (Pritchard et al., 2016). To estimate core and unique Clusters of Orthologous Groups (COG) of protein sets across the three species, COG profiles for each of the MAGs and SAGs were taken from JGI IMG and transformed into presence and absence. Microsoft Excel (=MAX function) was used to combine the presence and absence for each ANI-based species and (=COUNTIF function) to compare the three species. The Venn diagram was created using Python.

### Assessing DChs_GAL08 Abundance Using qPCR

The abundance of DChs_GAL08 was estimated *via* qPCR of 16S rRNA genes specific to DChs_GAL08. qPCR primers were designed using full-length DChs_GAL08 16S rRNA gene sequences (1,576 bp) extracted from the species representing the most abundant SAGs (e.g., 2616644824, Table 1). The full-length 16S rRNA gene sequence was aligned *via* parsimony into release 123 of the ARB-Silva database using the ACT: alignment, classification, and tree service online tool provided by ARB-Silva. Primers GAL08_842 (5′-TAGTCCCTCCGTGCCTGT-3′) and GAL08_992 (5′-GGCTACTACCCGCAGTTC-3′) were generated using the probe design function of ARB (Ludwig et al., 2004) and synthesized using Thermo Fisher Scientific (Waltham, MA, United States). Primers were tested on sediment containing varying relative abundances of GAL08 sequences, as determined by prior amplicon sequencing. Samples that contained no GAL08 sequences in amplicon sequencing analyses did not produce a detectable PCR product when amplified using the specific qPCR primers. Positive PCR products were cloned into the pJET1.2 vector using the Clone JET PCR Cloning Kit (Thermo Fisher Scientific). Sequences of two clones were confirmed using Sanger sequencing. qPCR reactions were set up using SYBR Green qPCR master mix (QIAGEN, Venlo, Netherlands). Inspection of qPCR melt curves, as well as testing on samples with no detected DChs_GAL08 using Illumina sequencing, suggested primer specificity. qPCR standards were generated by diluting a purified PCR product amplified with DChs_GAL08-specific primers from one of the clones. The reactions were run on a Rotor-Gene 6000 (QIAGEN, Venlo, Netherlands) using the following cycling conditions: an initial denaturation step at 95°C (10 min), 40 cycles at 95°C (10 s), 53°C (15 s), and 72°C (20 s), a pre-melt conditioning step at 72°C (90 s), and a melt ramp from 72 to 95°C increasing by 1°C every 5 s.

### Fluorescence *in situ* Hybridization on GAL08-Containing Sediment Samples

Samples from the Dewar Creek hot spring did not produce quality fluorescence *in situ* hybridization (FISH) images with any probes applied possibly because samples had been stored for several years beforehand. Therefore, fresh samples from the geochemically similar Hoodoo Creek hot spring in British Columbia, Canada, were used (GPS coordinates: 51°20′41″ and 125°37′21″; 83°C at the source, pH 7.3). Samples were collected in August 2021 along with the outflow of this spring at 61°C. The presence of GAL08 was confirmed using qPCR of 16S rRNA genes using DChs_GAL08-specific primers, as described in the previous section. Counts were estimated at 3.8 × 10^4^ gene copies per gram sediment. Samples were frozen at −80°C, then kept at 4°C for a few days before FISH.

Fluorescence *in situ* hybridization was carried out according to previously described methods (Amann, 1995) using two probes: GAL08_185_cy3 (yellow; 5′-/5Cy3/CTG TTG GTG AAA GCG GGG GAC-3′), specific to GAL08, and EUB338_Alexa488 probe (green; 5′-/Alexa488/GCT GCC TCC CGT AGG AGT-3′), specific to bacteria. FISH probe GAL08_185_cy3 was designed specifically for the 16S rRNA gene of DChs_GAL08. It had at least two mismatches to all non-target organisms (ARB TestProbe 3.0) and was computationally evaluated using mathFISH (Yilmaz et al., 2006, 2011). Optimization was carried out using different concentrations of formamide (20–40%, vol/vol) (Yilmaz and Noguera, 2004). The probes were commercially synthesized and 5′ labeled with the fluorescent dyes Cy3 (Integrated DNA Technologies, San Diego, CA, United States) and Alexa 488 (Invitrogen), respectively. *Escherichia coli* DH5α competent cells from the TOPO TA cloning kit (Thermo Fisher Scientific) were cultured overnight and used as positive controls for the EUB338-Alexa488 probe. Samples were diluted using 1 mL of phosphate-buffered saline (PBS) solution (0.1 M, pH 7.0), then fixed at 4°C for 1 h. Fixed cells were recovered using centrifugation at 10,000 × *g* for 2 min, washed twice using 1 mL of PBS, and recentrifuged. After the second wash, the fixed cells were resuspended in 300 μL of PBS/100% ethanol (1:1 v/v). For each microscope slide well, a 10 μL sample of PBS/ethanol cell suspension was immobilized, dehydrated, and permeabilized. Hybridization was carried out at 30% formamide concentration for 2 h at 46°C using 13 μL of hybridization buffer [0.82 M NaCl, 18 mM Tris–HCl, 0.009% (w/v) SDS, pH 7.2] and 1 μL of each probe per well. Samples were simultaneously hybridized with the GAL08_185_cy3 probe and the EUB338_Alexa488 probe at concentrations of 100 ng μL^–1^ and 50 ng μL^–1^, respectively. The cells were resuspended at 48°C for 15 min in 50 mL of wash buffer (0.1 M NaCl, 20 mM Tris–HCl, 5 mM EDTA, 0.01% SDS, pH 7.2). Microscopic slides were mounted using VECTASHIELD^®^ antifade mounting medium (Vector Laboratories, Burlingame, CA, United States) and kept at 4°C. Following FISH, the samples were observed using an Olympus BX51 microscope (Life Science Solutions, Toronto, ON, Canada) using a 100× oil immersion objective (UPlanFLN U152, Olympus). Cy3 and Alexa Fluors were excited using an X-Cite^®^ 120Q illumination system (Photonic Solutions, Mississauga, ON, Canada) using Cy3 and FITC filters, respectively. Images were captured using a QImaging Retiga 2000R FAST 1394 digital camera and microscopy imaging software ImagePro Express version 6.3 (Media Cy, MD, United States), then processed using ImageJ software (Schneider et al., 2012).

### Enrichment Cultures

Enrichment cultures were set up in triplicate using source water and sediment collected from the Dewar Creek hot spring in 2017. Source water was autoclaved before use and included as a nutrient source. Sediment was collected at 65°C and stored at 4°C for up to 16 months before enrichments were initiated. To create the inoculum, five samples high in GAL08 16S rRNA gene copy numbers (as determined using qPCR) were mixed in equal ratios under a flow of N_2_ gas to minimize exposure to O_2_. An aliquot of this mixed sediment was frozen for downstream qPCR and Illumina sequencing to obtain an exact concentration of DChs_GAL08 in the inoculating sediment. Each 11.5-mL enrichment vial contained 500 μL of mixed source sediment inoculum and 1,500 μL of source water. Vials were sealed with butyl rubber stoppers and capped with metal crimps. Treatments included amendment with a protein mix (protease peptone, protein hydrolyzate amicase, and yeast extract at a final concentration of 0.1 g L^–1^ each, or 0.3 g L^–1^ total in the enrichment vials), amendment with a carbohydrate mix (Supplementary Table 2), and unamended controls. The protein mix was sterilized by autoclaving, and the carbohydrate mix was sterilized as described in Supplementary Table 2. A protein mix was used because the DChs_GAL08 SAGs contained a large proportion of genes for amino acid metabolism based on COGs, which is not uncommon for *Acidobacteria* (Eichorst et al., 2018). To create an atmosphere of 5% v/v CO_2_ and 1% v/v O_2_ in N_2_, the enrichment vials were first evacuated for 4 min on a vacuum manifold and refilled with N_2_ (Praxair, 99.998%). Then, CO_2_ (Praxair, 99.5%) and ambient air were injected with a syringe to give final mixing ratios of roughly 5% CO_2_ and 1% O_2_. The decision to use a high pCO_2_ was guided by literature (Stevenson et al., 2004) and a low pO_2_ by the presence of denitrification genes in the SAG data, the hypoxic nature of the Dewar Creek hot spring water *in situ*, and previous experiments (Supplementary Table 3). All enrichment vials were incubated at 65°C, without shaking. Substrate-amended samples were allowed to equilibrate to 65°C for 44 h, then sampled for the time 0 point by removing 500 mL of a water/sediment slurry, and immediately freezing at −20°C for DNA extraction. Sampling was repeated at 2, 4, and 6 weeks. The initial atmospheres in the bottles were reconstituted every 3–4 days by repeating the N_2_ flush and injection of CO_2_ and O_2_ using a syringe. DNA extraction and qPCR were performed as described above.

### Sequence Data

Accession numbers for SAGs and MAGs analyzed in this study can be found in Table 1. Amplicon sequencing data can be found in the SRA repository under accession number PRJNA779083.

## Results

### DChs_GAL08 Are Thermophiles

**FIGURE 1:**
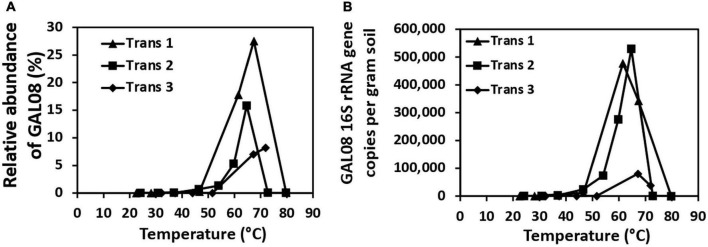
Relative abundances of DChs_GAL08 based on 16S rRNA gene amplicon sequencing **(A)** and qPCR counts of DChs_GAL08 16S rRNA gene copies per gram of sediment **(B)** in sediments along three temperature transects in the Dewar Creek hot spring.

Three temperature transects sampled in 2015 indicated that DChs_GAL08 is a thermophile with a temperature preference of 60–70°C, based on both amplicon relative abundance and absolute counts of DChs_GAL08-specific 16S rRNA genes (Figure 1). GAL08 was also detected in five out of six Dewar Creek sediments collected at temperatures between 22.5 and 32.2°C (Supplementary Figure 2); however, the very low relative abundance (0.01% or below) of GAL08 reads at these low temperatures makes it difficult to discern whether GAL08 is capable of growth at these temperatures or whether some cells are simply dispersing downstream from hotter upstream areas and not growing in the cooler areas. Takami et al. (2012) used G + C content of a GAL08 16S rRNA gene sequence (62.4%) to estimate the maximum growth of GAL08 at 86.7°C; however, we did not detect any GAL08 reads in two Dewar Creek sediments collected at 85.9°C, despite similarly high 16S rRNA gene sequence G + C content of the DChs_GAL08 (62.2%). Additionally, no genes encoding reverse gyrase (Pfam17915), a hyperthermophile-specific enzyme indicative of growth at or above 70°C, were found in any of the DChs_GAL08 SAGs, further supporting a more moderate temperature optimum for DChs_GAL08. The hottest sediment that produced detectable GAL08 reads at Dewar Creek was collected at 79.9°C.

To assess the presence of these bacteria in other environments, the IMNGS platform (Lagkouvardos et al., 2016) was used to search the Sequence Read Archive (SRA) (Wheeler et al., 2008) for closely matching 16S rRNA gene sequences. The query sequence was a complete 16S rRNA gene from Species 1 (Table 1). Sequences at >97% and >99% identity were retrieved. Most of the habitats where similar sequences were detected were thermal springs, along with a thermal oil reservoir (Supplementary Figure 3), although sequences were also detected less commonly in some mesic sites. Closer examination of the metadata for the thermal sites where DChs_GAL08 was detected (available for 11 out of 13 sites) showed that they were pH neutral to slightly alkaline (pH 5.9–9.0). Temperatures in these sites ranged from 37 to 110°C, but most (9 out of 12 sites with available temperature metadata) were between 55 and 81°C. In general, these bacteria seem to prefer sites similar in pH and temperature to the Dewar Creek hot spring. A search of the Genomes from Earth’s Microbiomes (GEM) database (Nayfach et al., 2021) also revealed that high-quality GAL08 MAGs were only binned from thermal spring environments. In addition to Dewar Creek sites, high-quality GAL08 MAGs were found in sediment from Great Boiling Spring and a freshwater sample taken near Shoshone Lake from Yellowstone National Park, with accessions 3300020139_8 and 3300005858_6, respectively.

Although FISH staining was unsuccessful with Dewar Creek samples (probably because of long storage), freshly collected sediment from a similar site, the Hoodoo Creek hot spring, was used for FISH. FISH images indicated that GAL08 are very short rods less than 1 μm in length and occur in biofilms with other bacteria and archaea (Supplementary Figure 4).

### DChs_GAL08 Is a Stable Component of the Dewar Creek Hot Spring Community

**FIGURE 2:**
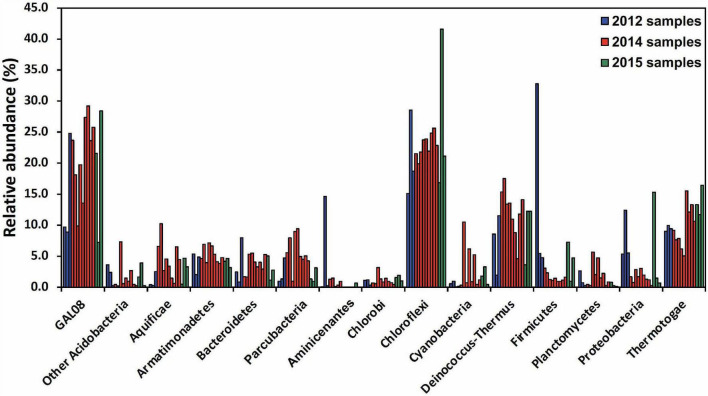
Amplicon-based community analysis of 15 samples collected between 64.3 and 67.4°C at the Dewar Creek hot spring during three sampling trips in 2012, 2014, and 2015. Multiple samples at unique locations were collected during each sampling trip and each sample is represented as a separate bar color-coded by year. Only bacterial phyla that were at or above 3% relative abundance in at least 1 of the 15 samples are included. The *Acidobacteria* are split into DChs_GAL08 and all other *Acidobacteria*. Relative abundance data were calculated based on 16S rRNA amplicon sequencing.

A community analysis of 15 samples collected between 64.3 and 67.4°C was performed to elucidate the typical microbial community at Dewar Creek hot spring near 65°C, the temperature optimum of DChs_GAL08. At this temperature, the microbial community (Figure 2) is dominated by OTUs belonging to *Chloroflexi*, *Acidobacteria*, *Deinococcus-Thermus*, and *Thermotogae*, all of which were commonly detected at relative abundances of 10% or higher. Other phyla typically detected at over 3% of relative abundance included *Armatimonadetes*, *Firmicutes*, Parcubacteria (OD1), *Proteobacteria*, *Bacteroidetes*, and *Aquificae*. All 15 samples collected between 64.3 and 67.4°C over different sampling years were positive for GAL08, with relative abundances ranging from 7.0 to 29.2% (Supplementary Figure 5). In all high-temperature samples (60–85°C), GAL08 was the dominant acidobacterium, making up on average 89.0% of all acidobacterial reads detected (Supplementary Figure 3).

### Diversity of DChs_GAL08

**FIGURE 3:**
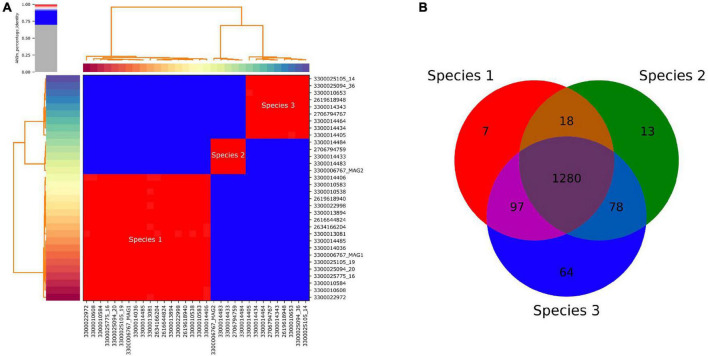
**(A)** Pairwise average nucleotide identity (ANI) of all recovered single amplified genomes (SAGs) and metagenome-assembled genomes (MAGs) of DChs_GAL08, showing 3 distinct species-level clusters. Genomes are identified using JGI taxon IDs. The three red clusters all show >99.2% identity, while the blue clusters all have <88.1% identity. The figure was calculated and drawn using pyani (Pritchard et al., 2016). **(B)** Venn diagram based on the presence or absence of COGs in the three species.

Average nucleotide identity comparisons of all SAGs and MAGs (Figure 3) revealed that they corresponded to three species, based on a species delineation of 95–96% ANI identity (Goris et al., 2007; Kim et al., 2014). One of these three species (Species 1 in Table 1 and Figure 3) was dominant, comprising 14 out of the 25 SAGs. Amplicon analysis also revealed three ASVs for the DChs_GAL08 group. The predominant ASV, ASV1, corresponded to SAG Species 1 (maximum 1 nucleotide error). ASV1 was dominant in 16S rRNA gene amplicons from samples taken at all temperatures (Supplementary Figure 6), making up on average 95.9% of reads identified as DChs_GAL08. ASV2 (Species 2) only exceeded 30% in 2 samples, while ASV3 was always very rare (Supplementary Figure 6).

The 16S rRNA genes of Species 1 and 3 differed by only 0.19% based on full-length sequences, while the difference between these two species and Species 2 was larger but did not exceed 1.21%. Core genome analysis (performed in IMG) showed that the vast majority of COGs present were shared across all three species, with only 51–64 COGs unique to each (Figure 3).

### GAL08 Is a Subgroup of *Acidobacteria*

**FIGURE 4:**
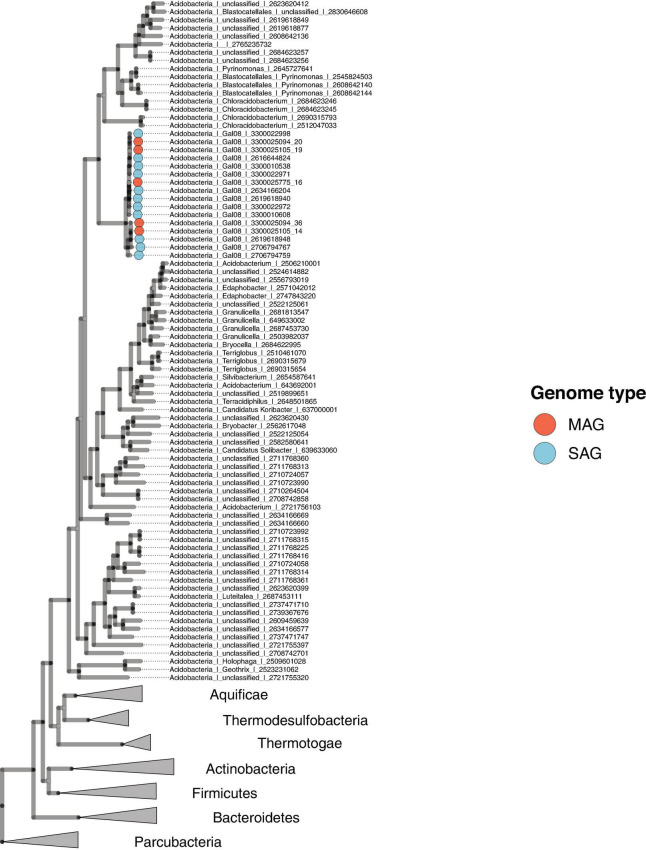
Maximum likelihood concatenated marker gene tree showing the phylogenetic placement of GAL08 within the domain *Bacteria*. The tree includes eleven GAL08 SAGs, five GAL08 MAGs, 68 *Acidobacteria* genomes, 214 genomes belonging to 6 other phyla, and 6 genomes from the *Parcubacteria*, serving as the outgroup. The 214 genomes were selected from a reduced set of bacterial isolate genomes available in the Integrated Microbial Genomes (IMG) database (Chen et al., 2021). The total set of (61,619) available genomes was first reduced by clustering the RNA polymerase beta-subunit gene at 65%; then, the reduced set was further minimized by screening for genomes belonging to phyla to which the GAL08 16S rRNA gene sequence shows the closest 16S rRNA gene identity (*Thermotogae*, *Aquificae*, *Thermodesulfobacteria*, *Firmicutes*, *Actinobacteria*, and *Bacteroidetes*, at 82–84%); the final set served as a reference database for downstream tree inference.

**FIGURE 5:**
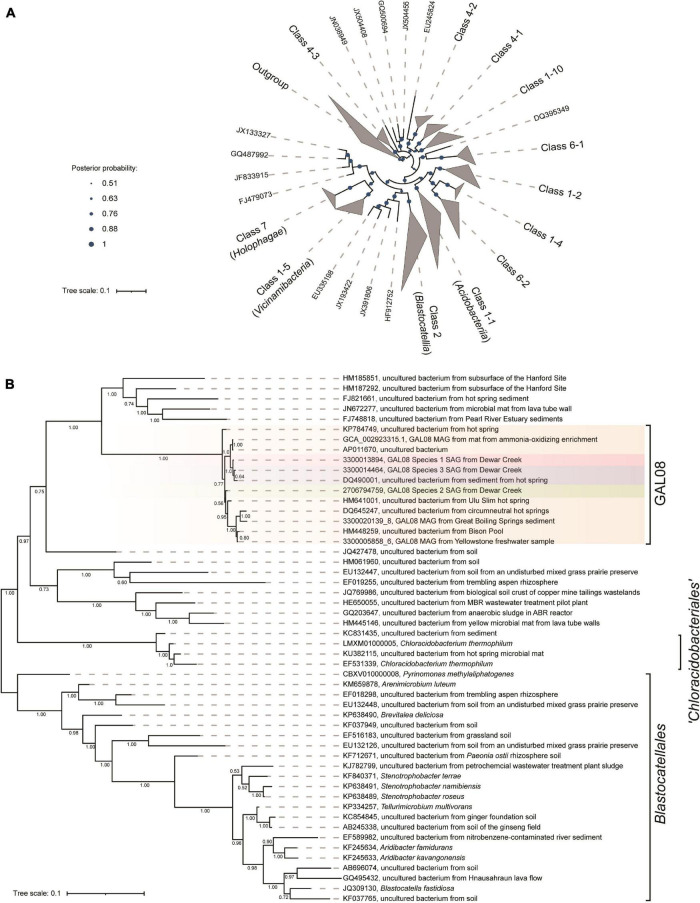
**(A)** Bayesian 16S rRNA gene tree built using the ARB-Silva.de SSU rRNA gene reference database, release 138, showing the relationship of the major subgroups within the *Acidobacteria*. Nomenclature for the subgroups is used as suggested by Dedysh and Yilmaz (2018). The outgroup contains one 16S rRNA gene sequence from an isolate in each of 29 cultivated phyla. Confidence values, indicating posterior probabilities, are depicted as circles of different sizes. The scale bar represents 0.1 nucleotide substitutions per nucleotide position. **(B)** An expansion of the GAL08 within the *Blastocatellia.* All of the GAL08 sequences available in release 138 of the ARB-Silva database are included, as well as representative 16S rRNA gene sequences from each of the three species represented by SAGs and MAGs in this study. Two MAGs that contained 16S rRNA gene sequences from two other sites covered by the dataset of Nayfach et al. (2021) were also included. The sequences selected for *Blastocatellia* include all isolates and 1 to 2 sequences from each major clade available in release 138 of the ARB-Silva database. A posterior probability of 1.00 strongly supports the separation of GAL08 from the other members of the order *Blastocatellales*. The scale bar represents 0.1 nucleotide substitutions per nucleotide position. The list of accession numbers used in the construction of this tree is provided in Supplementary Table 1.

A concatenated genome marker phylogeny including selected DChs_GAL08 SAGs and MAGs verifies the presence of three GAL08 species clusters within the class *Blastocatellia* (Figure 4). *Pyrinomonas* and *Chloracidobacterium* are the closest cultured genera with available genomes. To verify and refine this assignment, a 16S rRNA gene tree was constructed incorporating nearly full length (>1,395 bp) 16S rRNA gene sequences from across the breadth of the *Acidobacteria* (Figure 5). In this analysis, the GAL08 group clusters as a distinct subgroup separate from other members of the class *Blastocatellia*. Using the taxonomic boundaries suggested by Yarza et al. (2014), an 80.2% average 16S rRNA gene divergence to cultivated *Blastocatellia* species would place GAL08 as a new taxonomic order within this class.

### Metabolic Predictions for DChs_GAL08

**FIGURE 6:**
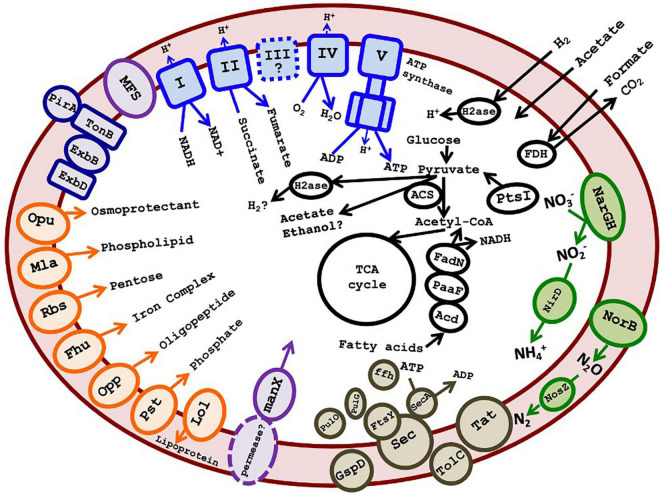
Cartoon depicting putative metabolism of GAL08 based on SAG analysis. Predicted metabolic components include an oxidative phosphorylation pathway, an incomplete denitrification pathway (absence of *nirKS*), acetate fermentation, fatty acid degradation, a small number of ABC transporters (shown in orange on the left of the figure), a PTS system for mannose, several major facilitator superfamily transporters, Sec-SRP plus TolC and Type II secretion systems, a TonB-dependent outer membrane transport system possibly acting in siderophore transport, and potential fermentation pathways. The symbol “?” and dashed lines both indicate a speculative metabolic component or product. A list of accession numbers for the genes shown is provided in Supplementary Table 4. A list of amino acid auxotrophies is shown in Supplementary Table 6.

The 16 most complete SAGs and the 7 MAGs are summarized in Table 1. These ranged in estimated completeness from 30% to 93%, with minimal contamination. The average genome size, estimated *via* CheckM (Parks et al., 2015), was 3.17 Mb (Table 1). The average G + C% of the SAGs was 62.8%, the same as the G + C content of the 16S rRNA gene. Species 1 included several high-quality genomes with 70–94% estimated completeness, suggesting that sufficient data were available for metabolic prediction. The 16 most complete DChs_GAL08 SAGs (Table 1) were analyzed together using the IMG platform, with reference to MetaCyc (Caspi et al., 2014) and Kyoto Encyclopedia of Genes and Genomes (KEGG) (Kanehisa and Goto, 2000) pathways. SAGs were preferred to MAGs for this because the high similarity of the three species could complicate compositional binning. A summary of some metabolic predictions is presented in Figure 6 and Supplementary Table 4.

Genes encoding outer membrane proteins such as OmpA and TolC and lipopolysaccharide export system permease LptF/LptG indicate that the bacterium is Gram-negative, while shape-determining proteins MreB, MreC, MreD, and RodA indicate a rod shape. FISH imaging verified that cells were very short rods, estimated at 0.8 μm in length. This is small compared with cultured species in the class *Blastocatellia*, such as *Blastocatella fastidiosa* (family *Blastocatellacea*, 0.8–0.9 μm × 0.8–12.0 μm) (Foesel et al., 2013), *P. methylaliphatogenes* (family *Pyrinomonadaceae*, 0.3–0.6 × 1–4 μm) (Lee et al., 2015), and *Brevitalea deliciosa* (family *Arenimicrobiaceae*, 0.6–0.8 × 0.9–1.8 μm) (Wüst et al., 2016).

No key genes involved in phototrophy were detected. This was verified using BLAST of key phototrophy genes found in *C. thermophilum* (Garcia Costas et al., 2012) against the SAGs. Common CO_2_ fixation pathways such as the Calvin-Benson-Bassham cycle were incomplete.

Genes encoding glycolysis, the TCA cycle, and oxidative phosphorylation (including NADH dehydrogenase, succinate dehydrogenase, some of the bc complex, and cytochrome c oxidase) were detected, indicating a capacity for aerobic respiration. Conversion of pyruvate and acetyl-CoA in glycolysis/gluconeogenesis occurs *via* a pyruvate:ferredoxin oxidoreductase (Pfr). Besides a single-component Pfr, there are also multiple 2-component 2-oxoglutarate ferredoxin oxidoreductases in individual SAGs (Supplementary Table 4). The presence of Pfr is an indication that the organism prefers low O_2_ tensions, as this enzyme may be damaged by exposure to O_2_ and is present primarily in anaerobes, whereas aerobes usually use pyruvate dehydrogenase to produce acetyl CoA (Chabrière et al., 1999; Khademian and Imlay, 2020). In addition, the TCA cycle appears to proceed *via* ferredoxin-dependent 2-oxoglutarate synthase rather than NAD^+^-dependent 2-oxoglutarate dehydrogenase. In general, the use of low-redox ferredoxin enzymes may indicate a preference for microaerophilic or anaerobic growth.

A facultatively anaerobic or microaerophilic lifestyle is in keeping with this hot, O_2_-poor habitat. Catalases were absent, but genes encoding O_2_ tolerance proteins such as peroxidases and Hsp33, a chaperone responding to oxidizing conditions, were found. The presence of hemerythrin-like proteins also suggests that DChs_GAL08 may have mechanisms for sensing, detoxifying, sequestering, or transferring O_2_ (Alvarez-Carreño et al., 2018). Alternatively, hemerythrin-like proteins could be used for nitric oxide sensing or tolerance (Alvarez-Carreño et al., 2018), and indeed some of the genes encoding hemerythrin-like proteins were found in gene clusters also encoding nitrate reductase and nitric oxide reductase (Supplementary Figure 7). Anaerobic respiratory capability is indicated by genes encoding an incomplete denitrification pathway, including dissimilatory nitrate, nitric oxide, and nitrous oxide reductases (Figure 6 and Supplementary Table 4). Two genes encoding dissimilatory reduction of nitrate to nitrite, *narG* and *narH*, were detected. While *narJ* and *narI* were not annotated, in two of the three species, the *narG* and *narH* genes are present directly upstream of genes annotated as encoding a DMSO-reductase chaperone and a DMSO-reductase heme (Supplementary Table 4 and Supplementary Figure 7), which could code for proteins with analogous functions to the chaperone NarJ and the heme-iron-containing *Nar*I, thereby completing the *narGHIJ* operon. Other anaerobic respiratory pathways were not found, although analysis using FeGenie (Garber et al., 2020) detected a homolog of *mtrC*, encoding a key outer membrane protein that transfers electrons to Fe^3+^ in the Mtr system of dissimilatory iron reducers. Other genes in the Mtr system (*mtrAB*) are not present, but the putative *mtrC* gene is adjacent to a cluster of genes encoding cytochrome c oxidase, suggesting that it is involved in electron transfer in some way.

Some evidence also exists for fermentation pathways. We note at the outset that many potential fermentation enzymes are multifunctional and/or bidirectional and have also been found in some other cultured acidobacteria that have not been shown to grow fermentatively (Eichorst et al., 2018). Therefore, fermentation should be considered as a potential function that requires further verification. Fermentation from pyruvate to acetate could potentially proceed *via* Pfr coupled to an AMP-forming acetyl CoA synthetase (Acs) (Figure 6), a pathway demonstrated in *Syntrophus aciditrophicus*, and hypothesized to operate in other bacteria (James et al., 2016). This pathway would require high pyrophosphate levels and a high AMP/ATP ratio, otherwise, the Acs would be unidirectional toward acetyl-CoA (Kumari et al., 2000; James et al., 2016). Regeneration of the reduced ferredoxin formed by Pfr during fermentation could occur *via* a ferredoxin:NADP^+^ oxidoreductase or a hydrogenase (see below). Alternative pathways of acetate formation are probably absent: there is no ADP-forming Acs (EC:6.2.1.13) used by organisms like *Pyrococcus furiosus* (Glasemacher et al., 1997). The pathway of acetate formation *via* acetyl-P using phosphate acetyltransferase plus acetate kinase is missing the latter enzyme, although an acylphosphatase could possibly substitute. Other potential fermentation products include ethanol (*via* acetaldehyde dehydrogenase and alcohol dehydrogenase) or H_2_ (hydrogenase) (Supplementary Table 4).

Genes encoding two components of a NiFe hydrogenase are encoded in all SAGs of Species 2 but are missing from Species 1 and 3. Instead of the third, cytB-containing component (VhoC) found in some characterized F420-non-reducing hydrogenases, the two structural genes are adjacent to a cyt-C encoding gene. Other genes in the same genomic region encode a heterodisulfide reductase that likely accepts electrons from the hydrogenase, a full set of hydrogenase maturation proteins, and two components of a four-component NADP-reducing iron-only hydrogenase (Supplementary Table 4 and Supplementary Figure 8). This region, summarized in Supplementary Figure 6, does suggest the presence of at least one unusual hydrogenase in Species 2. The presence of these genes on a large genomic island that is not present in the other species suggests that they may have been obtained *via* lateral gene transfer (LGT). Top BLAST hits of the translated genes against the RefSeq Protein database showed best hits (49–76% identities) to bacteria from several phyla, including *Cyanobacteria*, *Deltaproteobacteria*, *Thermotogales*, *Chloroflexi*, and other *Acidobacteria* (Supplementary Table 5).

Predicting the external energy substrates for the respiratory or fermentative metabolism of these bacteria is difficult. Potential substrates for respiration include small organic acids that should diffuse freely into the cell, such as formate (*via* formate dehydrogenase). Acetate and H_2_, discussed as potential fermentation products above, could in fact be energy-yielding substrates depending on the directionality of the Acs and the putative hydrogenase enzymes. Genes encoding several glycosyl hydrolases (family GH4, GH31, GH38, GH53, GH57, and GH43/DUF377), including genes encoding an alpha-glucosidase (GH31) and a beta-glucosidase (GH3), also suggest some capacity for sugar metabolism. However, the annotated transporters included only one predicted pentose sugar ABC transporter. The lack of other known ABC sugar transporters, including for glucose, suggests the possibility that the glycolysis pathway in DChs_GAL08 is primarily used for gluconeogenesis. However, of the four enzymes associated with gluconeogenesis (pyruvate carboxylase, glucose 6-phosphatase, phosphoenolpyruvate carboxykinase, and fructose 1,6-bisphosphatase), only genes encoding phosphoenolpyruvate carboxykinase (in Species 3 only) and fructose 1,6-bisphosphatase were detected. Other predicted ABC transporters were limited to osmoprotectant, phospholipid, oligopeptide, phosphate, and iron complex transporters. However, several major facilitator superfamily 1 transporters were present, as was an operon encoding all three components of a phosphotransferase (PTS) system. The IIA component of the PTS operon belongs to the “mannose” family, which typically interacts non-specifically with multiple hexoses, including glucose (Magoch et al., 2020). What permease the PTS system interacts with is unclear.

Apart from sugars, the presence of oligopeptide transporter encoding genes and nearly complete annotated valine, leucine, and isoleucine degradation pathways in all three species suggests an ability for protein scavenging. Indeed, a comparison of the 16 DChs_GAL08 SAGs in Table 1 with genomes of 69 cultured *Acidobacteria* available in IMG showed that they had a lower percentage of their genomes devoted to COG category G: carbohydrate transport and metabolism (average 5.10%, compared with 8.56% in other *Acidobacteria*), but a larger percentage devoted to category E: amino acid transport and metabolism (average 10.55%, compared with 8.47% in other *Acidobacteria*). This suggests that they may be more capable of metabolizing proteins than sugars. Fatty acid scavenging pathways are also complete and could provide Acetyl-Co and NADH for respiration. In summary, there are multiple possible energy substrates for these bacteria, but a firm conclusion about the primary energy sources will require culturing.

The SAGs contained genes annotated as involved in exopolysaccharide (EPS) biosynthesis. EPS biosynthesis genes are not unusual for *Acidobacteria* (Kielak et al., 2016) and could be used for adhesion and biofilm formation. Type II secretion system-encoding genes, *pulG* (pseudopilin), *pulO* (prepilin peptidase), and *GspG* (secretin), and a nearly complete Sec-SRP plus TolC secretion system were detected. These genes are sometimes implicated in virulence, but no other evidence for virulence was evident in the DChs_GAL08 SAGs. Genes encoding siderophore biosynthesis were not detected with FeGenie; however, multiple biopolymer/siderophore transport systems were detected (Guerinot, 1994). This suggests the possibility that DChs_GAL08 may scavenge siderophores from the environment. Siderophore transport genes have previously been reported for strains of Subgroups 1 and 3 (Ward et al., 2009) and Subgroup 4 (Lee et al., 2015) *Acidobacteria*. All three species of DChs_GAL08 are unable to synthesize biotin, and *bioY*, a biotin-specific transporter, was also detected in two of the three species. A list of proposed amino acid auxotrophies is shown in Supplementary Table 6.

### Laboratory Enrichments of DChs_GAL08

Enrichment cultures were set up to test some of the metabolic predictions described above. Although we failed to successfully produce an enrichment culture, some of these failures were illustrative.

Enrichment attempts under an air headspace suggested that DChs_GAL08 is sensitive to atmospheric levels of O_2_ (Supplementary Figure 9 and Supplementary Table 3). Using substrates such as trehalose, cellobiose, and benzoate under a full-air atmosphere resulted in an overgrowth of *Deinococcus-Thermus* from a relative abundance of 7.3% in the unincubated control to 42.5 and 78.3% in the incubated samples. These conditions resulted in a near elimination of detected GAL08 16S rRNA genes (*via* qPCR) in comparison to levels in the inoculating material (Supplementary Figure 9).

Incubations with various substrates under reduced O_2_ atmospheres containing 5% CO_2_ and 1% O_2_ in N_2_ gas performed better. When using mixed carbohydrates (Supplementary Table 6) or amino acids in the form of protease peptone and protein hydrolyzate amicase, initial declines of DChs_GAL08 were measured using qPCR in the first 2 weeks (Supplementary Figure 10). These were followed by rebounding populations in the latter 2–4 weeks of the cultivation. However, the substrate mixes did not result in a significantly higher population compared with the unamended controls, suggesting that residual substrates in inocula source water and sediment were supporting growth rather than the added substrates. The protein-amended samples showed a 3-fold increase over week 0 samples by the final week of sampling (week 6) (*t*-test, *p* = 0.0326) (Supplementary Figure 10). Although these experiments did not conclusively determine the growth substrates, they did demonstrate that growth was possible with the medium and the conditions provided and that microaerophilic conditions were preferred over aerobic conditions.

## Discussion

Microbial community analysis of sediment from the Dewar Creek hot spring revealed a high natural abundance of the uncultivated bacterial group, GAL08. DChs_GAL08 was a persistent member of the community, consistently detected in sediment around 65°C over 3 years of sampling. ANI comparisons of genomes obtained through single-cell sequencing revealed the coexistence of at least three species of DChs_GAL08, with Species 1 being dominant across all sampling years. GAL08 makes up around 90% of the acidobacterial community of the Dewar Creek hot spring at its preferred temperature (65°C) and over 95% at temperatures above 75°C (Supplementary Figure 3). Similar 16S rRNA gene sequences are detectable primarily in comparably hot (55–81°C), pH neutral thermal environments worldwide.

GAL08 sequences have been difficult to classify to a specific phylum in the past because of their high similarity to several different phyla. GAL08 16S rRNA genes show an 82–84% sequence similarity to 16S rRNA genes from bacteria belonging to nine different phyla: *Firmicutes*, *Thermodesulfobacteria*, *Thermotogae*, *Aquificae*, *Bacteroidetes*, *Actinobacteria*, *Proteobacteria*, *Nitrospirae*, and *Acidobacteria*. Additionally, the lack of research on this taxon leads to inconsistent naming in different classifiers: Greengenes, NCBI, EzTaxon, Genome Taxonomy Database (GTDB), Open Tree of life Taxonomy (OTT), and Silva. Phylogenetic placement of GAL08 varies in the literature. Takami et al. (2012) classified GAL08 as a sister phylum to *Acidobacteria*. Until as recently as release 123 of the ARB-Silva classifier also placed GAL08 as a phylum-level taxon (e.g., in the tree reported by Baker and Dick, 2013), but currently it places GAL08 sequences within the *Acidobacteria*; *Blastocatellia*; 11–24, where 11–24 is an uncultured order-level taxon. The GTDB (Parks et al., 2018) database also assigns sequences closely related to the DChs_GAL08, an uncultivated group called HR10, as an order within *Blastocatellia*. Our concatenated marker gene and 16S rRNA gene phylogenetic constructions agreed with the present consensus that GAL08 belongs to the phylum *Acidobacteria*, class *Blastocatellia*. The phylogenetic rank classifications proposed by Yarza et al. (2014) suggest that an 80.2% average 16S rRNA gene sequence divergence from cultivated *Blastocatellia* makes GAL08 an order-level group. Dedysh and Yilmaz (2018) delineated five potential orders within the *Blastocatellia*: *Blastocatellales*, “Chloroacidobacteriales”, Order 14-1, Order 14-2, and Order 24. GAL08, together with other bacteria in the 11–24 or HR10 clusters, belong to one of these candidate orders.

Analysis of SAGs indicated that the three species of GAL08 found at the Dewar Creek hot spring are capable of aerobic respiration, like other *Blastocatellia* (Ivanova et al., 2020), and are heterotrophic, like most *Acidobacteria* (Kielak et al., 2016). However, our SAG data also showed some evidence of anaerobic respiration and fermentation, indicating that they may be facultative anaerobes. While aerobic or microaerophilic species are more common among *Acidobacteria*, *Telmatobacter bradus* and *Paludibaculum fermentans* ferment sugars and polysaccharides (Pankratov et al., 2012; Kulichevskaya et al., 2014), *Geothrix fermentans* can ferment citrate or fumarate (Coates et al., 1999), and *T. aquaticum* can ferment pyruvate and proteins (Losey et al., 2013). Communities in samples from Dewar Creek sediment were a mixture of aerobic (such as *Synechococcus*) and anaerobic (such as *Anaerolineaceae*) organisms, suggesting that the sampling material, no more than 10 cm deep, spans the anoxic/oxic boundary. However, maintenance and growth of DChs_GAL08 were only observed under reduced O_2_ atmospheres.

In line with the analysis of Eichorst et al. (2018) of 24 complete acidobacterial genomes, an incomplete denitrification pathway was detected in DChs_GAL08. SAGs contained genes encoding NO_3_^–^, NO, and N_2_O reductases. None of the DChs_GAL08 SAGs, nor the genomes analyzed in the Eichorst et al. (2018) study, contained marker genes for dissimilatory sulfate reduction (*aprAB* and *dsrAB*). Genes encoding iron reduction were also not detected, with the single exception of a homolog of *mtrC*, which is common in *Acidobacteria*, based on a BLAST analysis of this gene. Homologs of all *mtr* genes are common in *Acidobacteria.* Several isolates, including *G. fermentans* (Coates et al., 1999), *T. aquaticum* (Losey et al., 2013), *P. fermentans* (Kulichevskaya et al., 2014), and *Acidobacterium capsulatum* (Coupland and Johnson, 2008), are capable of iron reduction.

While microaerophilic and anaerobic growth, therefore, both seem likely, the actual external substrates used for the growth of the DChs_GAL08 are not yet clear from either our genomic analyses or our enrichment cultures. Growth substrates could theoretically include organic acids, sugars, H_2_, or proteins. The dearth of transporters and the multifunctionality of key catabolic genes in the DChs_GAL08 genome data limited our ability to predict potential substrates. Compared with cultured *Acidobacteria*, more of the genome is devoted to amino acid transport and metabolism and less to carbohydrate transport and metabolism, possibly suggesting a preference for proteins. A recent analysis of all as-yet-published acidobacterial genomes illustrates that *Acidobacteria* can grow in different oxygen gradients, can utilize diverse carbohydrates, and can utilize both organic and inorganic nitrogen sources (Eichorst et al., 2018); the authors attribute the prevalence of *Acidobacteria* in environments with variable nutrient availability to the last two traits. This versatility, however, also imposes a challenge to enrichment efforts. Traditional approaches to culturing *Acidobacteria*, such as amendment with environmental extracts, a relatively low concentration of nutrients, amendment with complex polysaccharides, and relatively long incubation periods (Kielak et al., 2016) did not result in robust enrichment cultures of DChs_GAL08. Trying a less diverse carbohydrate mixture to minimize growth of competitors is worthwhile. Alternatively, incubating enrichments for longer than 6 weeks may result in population recovery significantly above initial levels; robustly growing cultures could then be amended with various substrates and tracked for changes in population density. Utilizing freshly collected sediment may also improve the probability of a successful laboratory enrichment.

The use of SAGs rather than MAGs allowed us to clearly identify some differences in the genetic makeup of the three closely related species of DChs_GAL08, without the potential for binning errors. These differences were consistent across multiple SAGs of each species and suggest some key differences among them, notably in catabolic potential. Catabolic modules that differed among the three included some glycosyl hydrolases present only in Species 1 or 2 and a large genomic island apparently encoding hydrogenase that was present only in Species 2.

In summary, we have documented a thermophilic and microaerophilic or facultatively abaerobic group of *Acidobacteri*a. At the Dewar Creek hot spring, GAL08 is not only the dominant clade of *Acidobacteria* but at 65°C is also often the most abundant member of the microbial community. We suggest the following putative name for this order: *Ca.* Frugalibacteriales: from L. adj. frūgālis: thrifty, frugal, simple; and propose the name *Ca.* Frugalibacterium for the candidate genus identified in this study. These bacteria are short rods. They are microaerophilic heterotrophs capable of aerobic respiration and potentially also fermentation and incomplete denitrification. Preferred habitats are pH-neutral thermal springs around 60–70°C. The cellular G + C% content is 62.8%, They belong phylogenetically to the phylum *Acidobacteria*, class *Blastocatellia.*

## Data Availability Statement

SAG and MAG data analyzed in this study are publicly available in the IMG database, under the taxon IDs indicated in Table 1. Amplicon sequencing data can be found in the SRA repository under accession number PRJNA779083.

## Author Contributions

IR performed sample collection, sample processing, SAG data analysis, qPCR, and enrichment culturing, and was a major contributor to the writing of the manuscript. JJ performed single-cell sorting and SAG creation. RB built the maximum likelihood-concatenated marker gene tree. CF assisted in enrichment culturing, qPCR, and FISH. SK and CF performed FISH staining. AS and TT performed some bioinformatic analyses. SG characterized the Dewar Creek hot spring and led multiple sampling expeditions to the site. TW oversaw single-cell sorting and SAG creation. PD contributed to experimental design and was a major contributor to the writing of the manuscript. All authors contributed to the article and approved the submitted version.

## Conflict of Interest

The authors declare that the research was conducted in the absence of any commercial or financial relationships that could be construed as a potential conflict of interest.

## Publisher’s Note

All claims expressed in this article are solely those of the authors and do not necessarily represent those of their affiliated organizations, or those of the publisher, the editors and the reviewers. Any product that may be evaluated in this article, or claim that may be made by its manufacturer, is not guaranteed or endorsed by the publisher.

## Footnotes

1
https://blast.ncbi.nlm.nih.gov

2
http://hmmer.org/

3
https://github.com/widdowquinn/pyani
