# Supplementary material for: GAL08, an Uncultivated Group of Acidobacteria, Is a Dominant Bacterial Clade in a Neutral Hot Spring
Source: Front Microbiol. 2022 Jan 11;12:787651. doi: 10.3389/fmicb.2021.787651 (PMC8787282; doi:10.3389/fmicb.2021.787651)
Supplement: Supplementary file 1 [file Data_Sheet_1.pdf]

Supplementary Table S1
A list of sequences included in the construction of the 16S rRNA gene sequence tree presented in Figure 5. The ARB-Siva.de release 132 accession number, full name, and isolation source, where available, is provided for each sequence used. We used all 12 database entries classified as GAL08 in release 132 of the Silva rRNA gene database (listed in the table below). Additionally, six full-length 16S rRNA gene sequences (obtained from SAG and MAG data) of three GAL08 species from the Dewar Creek hot spring were imported into the database and used in construction of this tree. The outgroup was created by selecting 1 sequence from each of the other 29 phyla with cultivated representatives.

Accession Number	Full Name	Source
AUAL01000024	*Acholeplasma axanthum ATCC 25176*	N/A
LLEU01000014	*Acidobacteria bacterium* OLB17	partial-nitritation anammox (PNA) reactor
CP001472	*Acidobacterium capsulatum* ATCC 51196	N/A
AB545935	*Actinomyces oris*	N/A
AP012029	*Anaerolinea thermophila* UNI-1	N/A
KF245633	*Aridibacter kavangonensis*	soil
EU136683	*Bacteroides pyogenes*	N/A
JQ309130	*Blastocatella fastidiosa*	soil
AANZ01000021	*Blastopirellula marina* DSM 3645	N/A
AM162405	*Bryobacter aggregatus*	*Sphagnum* peat bog
FR666706	*Bryocella elongata*	*Sphagnum* peat bog
AP012051	*Caldisericum exile* AZM16c01	N/A
CP000051	*Chlamydia trachomatis* A/HAR-13	N/A
CP002514	*Chloracidobacterium thermophilum* B	thermal spring
CP001100	*Chloroherpeton thalassium* ATCC 35110	N/A
X81319	*Chrysiogenes arsenatis* DSM 11915	N/A
AM749780	*Chthonomonas calidirosea*	steam-affected geothermal soil
AP011529	*Deferribacter desulfuricans* SSM1	N/A
AJ585986	*Deinococcus marmoris*	N/A
ABTR02000001	*Dethiosulfovibrio peptidovorans* DSM 11002	corroding offshore oil well
CP001146	*Dictyoglomus thermophilum* H-6-12	N/A
DQ528761	*Edaphobacter aggregans*	temperate soil
AM490846	*Elusimicrobium minutum*	N/A
CU928158	*Escherichia fergusonii* ATCC 35469	N/A
CP001792	*Fibrobacter succinogenes* subsp. succinogenes S85	N/A
AFQD01000460	*Fusobacterium nucleatum* subsp. animalis ATCC 51191	urogenital tract, vaginal
AP009153	*Gemmatimonas aurantiaca* T-27	activated sludge for phosphorus removal
AUAU01000033	*Geothrix fermentans* DSM 14018	N/A
AB372855	*Haloferula helveola*	N/A

X77215	*Holophaga foetida*	N/A
AB026268	*Hydrogenobacter subterraneus*	N/A
X82558	*Nitrospira moscoviensis*	N/A
AM749787	*Pyrinomonas methylaliphatogenes*	steam-affected geothermal soil
AF357916	*Sphaerochaeta globosa str. Buddy*	N/A
AB076610	*Sporotomaculum syntrophicum*	N/A
KP638491	*Stenotrophobacter namibiensis*	soil
KP638489	*Stenotrophobacter roseus*	soil
KF840371	*Stenotrophobacter terrae*	soil
KP334257	*Tellurimicrobium multivorans*	soil
HM214537	*Terriglobus saanen*sis SP1PR4	N/A
JF346116	*Thermosulfurimonas dismut*ans	deep-sea sulfidic structure at Mariner hydrothermal vent field
CP000916	*Thermotoga neapolitana* DSM 4359	N/A
GQ984352	uncultured bacterium	Northern Bering Sea surface water
AB128876	uncultured bacterium	deep marine sediments
AB238786	uncultured bacterium	N/A
AB240262	uncultured bacterium	N/A
AB672246	uncultured bacterium	rice paddy field soil
AB754130	uncultured bacterium	lake water
AB803523	uncultured bacterium	ocean drilling core sample
AB821092	uncultured bacterium	soil of Gotjawal Forest
AP011670	uncultured bacterium	N/A
DQ394896	uncultured bacterium	harbor sediment
DQ395349	uncultured bacterium	deep-sea octacoral
DQ490001	uncultured bacterium	sediment from hot spring
DQ645247	uncultured bacterium	circumneutral hot springs
EF492957	uncultured bacterium	soil around iron-manganese nodule
EF492968	uncultured bacterium	soil around iron-manganese nodule
EF580960	uncultured bacterium	freshwater calcareous mat
EU043668	uncultured bacterium	Altamaha and Ohopee River Valley
EU245824	uncultured bacterium	hypersaline microbial mat
EU328008	uncultured bacterium	moderate saline soil
EU335198	uncultured bacterium	saturated C horizon soil aggregate
EU487939	uncultured bacterium	sedment from Thalassia sea grass bed
EU669607	uncultured bacterium	soil
EU881247	uncultured bacterium	serious disturbance field soil
FJ269282	uncultured bacterium	sponge tissue

FJ478609	uncultured bacterium	undisturbed tall grass prairie
FJ478644	uncultured bacterium	undisturbed tall grass prairie
FJ478679	uncultured bacterium	undisturbed tall grass prairie
FJ479073	uncultured bacterium	undisturbed tall grass prairie
FJ479110	uncultured bacterium	undisturbed tall grass prairie
FJ538138	uncultured bacterium	paddy field soil
FJ545586	uncultured bacterium	North Yellow Sea sediment
FJ624910	uncultured bacterium	boreal pine forest soil
FJ624929	uncultured bacterium	boreal pine forest soil
FJ748818	uncultured bacterium	Pearl River Estuary sediments
FJ821661	uncultured bacterium	hot spring sediment
FN870304	uncultured bacterium	acidic coal mine lake
GQ487992	uncultured bacterium	soil polluted by heavy metals
GQ500694	uncultured bacterium	sandy clastic sediments
GQ500851	uncultured bacterium	bio-sep beads
GU180169	uncultured bacterium	sediment and soil slurry
HF912752	uncultured bacterium	microbial community of a marine sponge
HM185851	uncultured bacterium	subsurface of the Hanford Site
HM187292	uncultured bacterium	subsurface of the Hanford Site
HM243842	uncultured bacterium	Honghu Lake sediment
HM243859	uncultured bacterium	Honghu Lake sediment
HM445340	uncultured bacterium	mat from lava tube walls
HM445421	uncultured bacterium	mat from lava tube walls
HM448259	uncultured bacterium	Bison Pool
HM641001	uncultured bacterium	Ulu Slim hot spring
HQ121145	uncultured bacterium	loamy sand from a field
HQ445759	uncultured bacterium	deep saprolite and saprock
HQ445780	uncultured bacterium	deep saprolite and saprock
HQ597234	uncultured bacterium	grassland soil
HQ645194	uncultured bacterium	soil from Tibet Plateau
HQ674368	uncultured bacterium	subarctic Pacific Ocean
JF344017	uncultured bacterium	hydrocarbon polluted marine sediments
JF747659	uncultured bacterium	hypersaline groundwater
JF833915	uncultured bacterium	potassium mine soil
JN023646	uncultured bacterium	temperate highland grassland
JN038695	uncultured bacterium	Chongxi wetland soil
JN038949	uncultured bacterium	Chongxi wetland soil
JN038970	uncultured bacterium	Chongxi wetland soil
JN178091	uncultured bacterium	Texcoco lake extreme saline-alkaline soil

JN178438	uncultured bacterium	Texcoco lake extreme saline-alkaline soil
JN254798	uncultured bacterium	rhizospheric sludge
JN494156	uncultured bacterium	Guerrero Negro Hypersaline Mat
JN559123	uncultured bacterium	soil
JN672277	uncultured bacterium	microbial mat from lava tube wall
JN869187	uncultured bacterium	lake water
JN886858	uncultured bacterium	South West Indian Ridge carbonate sediments
JN977149	uncultured bacterium	Jiaozhao Bay sediment
JN977211	uncultured bacterium	Jiaozhao Bay sediment
JN977256	uncultured bacterium	Jiaozhao Bay sediment
JN995378	uncultured bacterium	anaerobic bioreactor fed with butyrate
JQ612184	uncultured bacterium	N/A
JQ612214	uncultured bacterium	N/A
JQ711709	uncultured bacterium	pothole sediments on arenitic sandstone
JQ825020	uncultured bacterium	saline-alkali soil
JQ941812	uncultured bacterium	diseased leaf
JX016641	uncultured bacterium	marine bulk water
JX133327	uncultured bacterium	diseased soil
JX193422	uncultured bacterium	intertidal surface sediment
JX206587	uncultured bacterium	marine sponge
JX221863	uncultured bacterium	subsurface aquifer sediment
JX222022	uncultured bacterium	subsurface aquifer sediment
JX222027	uncultured bacterium	subsurface aquifer sediment
JX222875	uncultured bacterium	subsurface aquifer sediment
JX227478	uncultured bacterium	Clarion-Clipperton Fracture Zone sediment
JX227517	uncultured bacterium	Clarion-Clipperton Fracture Zone sediment
JX391806	uncultured bacterium	marine sediment
JX504408	uncultured bacterium	oolitic sand
JX504455	uncultured bacterium	oolitic sand
KC255253	uncultured bacterium	calcium carbonate
KC331544	uncultured bacterium	apple orchard
KF037620	uncultured bacterium	soil
KF741569	uncultured bacterium	Talbert salt marsh sediment
KJ191789	uncultured bacterium	Pinus massoniana soil
KJ461654	uncultured bacterium	littoral wetland of a boreal lake
KJ590596	uncultured bacterium	membrane coupon in bioreactors
KM071728	uncultured bacterium	deep-sea hydrothermal vent sediments

KM221425	uncultured bacterium	hot spring
KP784749	uncultured bacterium	hot spring
KT361125	uncultured bacterium	gold mine
HM038000	*Vampirovibrio chlorellavorus*	N/A
AY049713	*Victivallis vadensis*	N/A
GCA_002923315.1	GAL08 MAG	mat from ammonia-oxidizing enrichment
3300014464	GAL08 Species 3 SAG	Dewar Creek
3300020139_8	GAL08 MAG	Great Boiling Springs sediment
3300005858_6	GAL08 MAG	Yellowstone freshwater sample
2706794759	GAL08 Species 2 SAG	Dewar Creek
3300013894	GAL08 Species 1 SAG	Dewar Creek

Supplementary Table S2 Substrates included in a carbohydrate mixture that failed to produce an enrichment of GAL08[a].

Substrate[b]	Sterilization Method	Rationale (predicted function in SAG/MAG analyses)
xylose	filtration	pentose transporter
arabinose	filtration	pentose transporter
sucrose	filtration	alpha glucosidase
maltose	filtration	alpha glucosidase
mannose	filtration	phosphotransferase system
locust bean gum	autoclaved	glycosyl hydrolases
mannan	filtration	glycosyl hydrolases
xylan	filtration	glycosyl hydrolases
dextran	filtration	glycosyl hydrolases
chitosan	autoclave	gluconase
chitin	autoclave	gluconase
N-acetyl glucosamine	filtration	gluconase
carboxymethyl cellulose	autoclave	gluconase

[a] Substrates were tested in two enrichment experiments, each set up in triplicate.
[b] All substrates were mixed at a final concentration of 0.1 g/L total substrate in the enrichment vials; carbohydrates were added in equal ratios, accounting for carbon number of substrates.

Supplementary Table S3. Relative abundance of detected Illumina 16S rRNA gene reads classified as either GAL08, *Deinococcus-Thermus*, or *Rhodothermus* when incubated at 65°C for one week with various substrates under normal atmospheric conditions. Relative abundance data was calculated from OTU tables produced by running QIIME pipeline version 1.9.1 (Caporaso et al., 2010); sequencing depth, represented by the total number of reads detected, is also indicated. Regardless of substrate used, incubation with atmospheric levels of oxygen resulted in a dramatic reduction in GAL08 16S rRNA relative abundance, while aerobic clades such as *Deinococcus-Thermus* and *Rhodothermus* increased in relative abundance. qPCR analysis of DChs_GAL08 16S rRNA gene reads for this experiment is shown in Supplementary Figure S8.

**Sample**	**Oxygen**	**Temp. (°C)**	**GAL08**	***Deinococcus -Thermus***	***Rhodothermus***	**Total reads**
Unincubated control[a]	Native	4	20.5 %	7.2 %	9.0 %	6018
Sodium benzoate	Atmospheric	65	0.0 %	59.4 %	13.3 %	2404
Cellobiose	Atmospheric	65	0.1 %	73.2 %	14.6 %	907
Trehalose dehydrogenase	Atmospheric	65	0.1 %	42.5 %	43.5 %	805
No substrate control	Atmospheric	65	0.1 %	78.3 %	6.8 %	2413

[a]The original sediment sampled from the Dewar Creek hot spring, stored at 4°C, that was used as inoculum for incubations at 65°C with the carbon sources shown.

Supplementary Table S4. Genes discussed in the text and detection in SAGs of each of the 3 species. An X indicates the gene was detected, a dash that it was not. Accession numbers are given, for one SAG only, where possible from the most complete SAG 3300013894. Where multiple genes are present in this SAG, all are listed. EC numbers or pfams were used for searching SAGs, and annotations were manually inspected in the case of enzymes with multiple pfams or multiple components.

Annotation	Sp1	Sp2	Sp3	Representative Locus
**Structural**				
OmpA family, pfam00691	X	X	X	Ga0170566_10150, Ga0170566_10268 Ga0170566_10586
OEP, outer membrane efflux protein (TolC), pfam02321	X	X	X	Ga0170566_101117, Ga0170566_101215 Ga0170566_10479, Ga0170566_10969, Ga0170566_11536
Lipopolysaccharide export system permease LptF/LptG, pfam03739	X	X	-	Ga0170566_10130, Ga0170566_10131
MreB/Mbl protein, pfam06723	X	X	X	Ga0170566_10844, Ga0170566_12324, Ga0170566_12624
rod shape-determining protein MreC, pfam04085	X	X	X	Ga0170566_12323
rod shape-determining protein MreD, pfam04093	X	X	-	Ga0170566_12322
rod shape determining protein RodA, pfam01098	X	X	X	Ga0170566_12320
**Anaerobic respiration**				
nitrate reductase alpha subunit, NarG, pfam 000384	X	X	X	Ga0170566_11174
nirate reductase beta subunit, NarH, pfam13247	X	X	X	Ga0170566_11172
DMSO reductase family type II enzyme chaperone pfam02613	X	X	X	Ga0070090_14744
DMSO reductase family type II enzyme, heme b subunit pfam09459	X	X	X	Ga0070090_14743
nitric oxide reductase subunit B, NorB, pfam00115	X	X	X	Ga0170566_11855
nitric oxide reductase NorQ protein, pfam07728, pfam08406	X	X	X	Ga0170566_12334
nitric oxide reductase activation protein, pfam 00092	X	X	X	Ga0170566_12333
NnrS protein/protein of unknown function, pfam04950	X	X	X	Ga0170566_11853
nitrous oxide reductase apoprotein, NosZ, pfam13473	X	X	X	Ga0070318_10983
nitrous oxidase accessory protein, NosD, pfam05048	X	X	X	Ga0170566_10846
decaheme c-type cytochrome, OmcA/MtrC family, pfam09699	X	X	X	Ga0170566_13014
**Key ferredoxin-using enzymes**				
Pfr, pyruvate ferredoxin/flavodoxin oxidoreductase, EC:1.2.7.1	X	X	X	Ga0170566_11217
2-oxoglutarate ferredoxin oxidoreductase subunit alpha, EC:1.2.7.3; EC:1.2.7.11	X	X	X	Ga0170566_102246, Ga0170566_1019, Ga0170566_10476
2-oxoglutarate ferredoxin oxidoreductase subunit beta, EC:1.2.7.3, EC:1.2.7.11	X	X	X	Ga0170566_102245, Ga0170566_1018, Ga0170566_10475
2-oxoglutarate ferredoxin oxidoreductase subunit delta EC:1.2.7.3; pfam12838	X	X	X	Ga0170566_10110
2-oxoglutarate ferredoxin oxidoreductase subunit gamma, EC:1.2.7.3; pfam01558	X	X	X	Ga0170566_1017
**Fermentation**				
acetyl CoA synthetase, Acs1, EC:6.2.1.1	X	X	X	Ga0170566_12628
FNR, ferredoxin:NADP+ oxidoreductase, EC:1.18.1.2	X	X	X	Ga0170566_10248
phosphate acetyltransferase, EC:2.3.1.8	X	X	X	Ga0170566_10393
acylphosphatase, EC:3.6.1.7	X	X	X	Ga0170566_10486
acetaldehyde dehydrogenase, EC 1.2.1.10	-	X (1/4)	X(3/4)	Ga0170557_10885
NAD(P)-dependent dehydrogenase, short-chain alcohol dehydrogenase family pfam13561	X	X	X	Ga0170566_101268, Ga0170566_11227

NAD(P)-dependent dehydrogenase, short-chain alcohol dehydrogenase family pfam00106	X	X	X	Ga0170566_10926, Ga0170559_10424
**Putative Hydrogenase (only present in Species 2)**				
NiFe hydrogenase small subunit (HydA, VhoG) pfam01058 EC:1.12.99	-	X (3/4)	-	Ga0070308_101430
NIFe hydrogenase large subunit (HydB, VhoA) pfam00374 EC:1.12.99	-	X (3/4)	-	Ga0070308_101431
cytochrome c554 and c-prime (pfam13435, pfam14537)	-	X (3/4)	-	Ga0070308_101429
NiFe hydrogenase iron-sulfur subunit D pfam02662 EC:1.12.99	-	X (4/4)	-	Ga0070308_101426
heterodisulfide reductase subunit A EC:1.8.98.1	-	X (4/4)	-	Ga0070308_101425
hydrogenase maturation protease pfam01750	-	X (3/4)	-	Ga0070308_101432
hydrogenase Ni incorporation protein HypA/HybF pfam01155	-	X (4/4)	X	Ga0070308_101433
hydrogenase nickel incorporation protein HypB pfam02492	-	X (2/4)	-	Ga0070308_101434
hydrogenase maturation protein HypF (pfam00708, pfam01300, pfam07503)	-	X (3/4)	-	Ga0070308_101435
hydrogenase maturation protein HypC pfam01455	-	X (3/4)	-	Ga0070308_101437
hydrogenase maturation protein HypD pfam01924	-	X (3/4)	-	Ga0070308_101438
hydrogenase maturation protein, carbamoyl dehydratase HypE (pfam00586, pfam02769)	-	X (2/4)	-	Ga0070308_101439
NADP-reducing hydrogenase subunit HndB EC:1.12.1.3	-	X (4/4)	-	Ga0070308_101418
NAD(P)-dependent iron-only hydrogenase diaphorase component flavoprotein HndC, EC:1.12.1.3	-	X (4/4)	-	Ga0070308_101419
**O_2 tolerance**				
Cytochrome c peroxidase, EC:1.11.1.5	X	X	X	Ga0170566_102126, Ga0170566_12050
thiol peroxidase, atypical 2-Cys peroxiredoxin EC:1.11.1.15	X	X	X	Ga0170566_11020
alkylhydroperoxidase/carboxymuconolactone decarboxylase family protein YurZ pfam02627	X	X	X	Ga0170566_11058, Ga0170566_12126
molecular chaperone Hsp33, pfam01430	X	X	X	Ga0170566_1282
Hemerythrin HHE cation binding domain-containing protein pfam01814	X	X	X	Ga0170566_10111
Hemerythrin-like domain-containing protein pfam01814	X	X	X	Ga0170566_101178, Ga0170566_1082, Ga0170566_11175, Ga0170566_11851, Ga0170566_11852
**Carbohydrate transport and metabolism**				
formate dehydrogenase iron-sulfur subunit pfam13247	X	X	X	Ga0170566_10595
Cytochrome b subunit of formate dehydrogenase pfam01292	X	X	X	Ga0170566_1306
Formate dehydrogenase assembly factor FdhD, pfam02634	X	-	X	Ga0170566_102152
Alpha-glucosidase (GH31), EC:3.2.1.20	X	X	X	Ga0170566_10965
Beta-glucosidase, EC:3.2.1.21 **(Species 1 only)**	X (4/7)	-	-	Ga0170566_10965
Family 4 glycosyl hydrolase, pfam02056	X	X	X	Ga0170566_102191
Glycosyl hydrolase family 53, pfam07745 (**Species 2 only)**	-	X (3/4)	-	Ga0070308_100537
Glycosyl hydrolase family 57, pfam03065	X	X	X	Ga0170566_10868
O-Glycosyl hydrolase (GH30, GH39), pfam01229, pfam02055, pfam17189 (**Species 2 only)**	-	X (4/4)	-	Ga0070308_1001142
predicted GH43/DUF377 family glycosyl hydrolase pfam04041	X	X	X	Ga0170566_11470, Ga0170566_11471
ribose transport system ATP-binding protein, EC:3.6.3.17	X	X	X	Ga0170566_102200
ribose transport system substrate-binding protein, pfam13407	X	X	X	Ga0170566_102203

ribose transport system permease protein, pfam02653	X	X	X	Ga0170566_102201
phosphoenolpyruvate carboxylase, type 1, EC:4.1.1.31 (**Species 3 only)**	-	-	X (3/5)	Ga0070318_100325
phosphoenolpyruvate carboxykinase (GTP) pfam00821	X	X	X	Ga0170566_10635
fructose 1,6-bisphosphate aldolase/phosphatase EC:4.1.2.13 pfam01950	X	X	X	Ga0170566_10386
oligopeptide transport system substrate-binding protein OppA pfam00496	X	X	X	Ga0170566_10237
oligopeptide transport system permease protein OppC OppD, pfam00528	X	X	X	Ga0170566_10236, Ga0170566_10235
peptide/nickel transport system ATP-binding protein/oligopeptide transport system ATP-binding protein OppD OppF pfam00005 pfam08352	X	X	X	Ga0170566_1152, Ga0170566_1153
phosphotransferase system, PTS-HPr, pfam00381	X	X	X	Ga0170566_105100
phosphotransferase system, enzyme I, PtsI EC:2.7.3.9	X	X	X	Ga0170566_10599
PTS system, mannose-specific IIA component EC:2.7.1.69	X	X	X	Ga0170566_105101
Major facilitator transporter MFS1, pfam07690	X	X	X	Ga0170566_102230, Ga0170566_102230, Ga0170566_11624, Ga0170566_1274
**Attachment**				
Uncharacterized protein involved in exopolysaccharide biosynthesis pfam13614 pfam13807 pfam02706	X	X	X	Ga0170566_104107
polysaccharide export outer membrane protein pfam02563 pfam10531	X	X	X	Ga0170566_104108
Type II secretory pathway, pseudopilin PulG; pfam07963	X	X	X	Ga0170566_10264, Ga0170566_10779
Prepilin signal peptidase PulO (type II secretory pathway) pfam06750 pfam01478	X	X	X	Ga0170566_10349
Type II secretion system protein G (GspG) pfam08334	X	X	X	Ga0170566_10649
Type II secretory pathway component GspD/PulD (secretin) pfam00263	X	X	X	Ga0170559_104116
Type II secretion system (T2SS), protein M subtype b pfam10741	X	X	X	Ga0256648_101311
**Siderophore transport**				
TonB C terminal, pfam13103, pfam03554	X	X	X	Ga0170566_10152, Ga0170566_106119
biopolymer transport protein TolQ/ExbB, pfam01618	X	X	X	Ga0170566_10154, Ga0170566_10620, Ga0170566_106118
biopolymer transport protein TolR/ExbD, pfam02472	X	X	X	Ga0170566_10153, Ga0170566_10621, Ga0170566_10622, Ga0170566_106116, Ga0170566_106117
iron complex outer membrane receptor protein/vitamin B12 transporter/ potential PirA-family-siderophore-receptor, pfam13620 pfam07715	X	X	X	Ga0170566_1213
iron complex transport system ATP-binding protein, EC:3.6.3.4	X	X	X	Ga0170566_1212
iron complex transport system permease protein, pfam01032	X	X	X	Ga0170566_1211
**Biotin (Species 1 only)**				
biotin transport system substrate-specific component (BioY))	X	-	-	Ga0170566_11190

Supplementary Table S5. Top BLASTP hits to the RefSeq protein database for the putative hydrogenase-encoding genes identified in Table S4.

**Putative Hydrogenase (only present in Species 2)**	Locus Tag	Top Blast hit (reference proteins only)
NiFe hydrogenase small subunit (HydA, VhoG) pfam01058 EC:1.12.99	Ga0070308_101430	76% *Candidatus* Sulfopaludibacter sp. (*Acidobacteria*) SbA4
NIFe hydrogenase large subunit (HydB, VhoA) pfam00374 EC:1.12.99	Ga0070308_101431	74% *Dehalogenimonas lykanthroporepellens* (*Chloroflexi*)
cytochrome c554 and c-prime (pfam13435, pfam14537)	Ga0070308_101429	47% *Kosmotoga pacifica* (*Thermotogales*)
NiFe hydrogenase iron-sulfur subunit D pfam02662 EC:1.12.99	Ga0070308_101426	74% *Dehalogenimonas lykanthroporepellens* (*Chloroflexi*)
heterodisulfide reductase subunit A EC:1.8.98.1	Ga0070308_101425	62% *Candidatus* Sulfopaludibacter sp. SbA4 (*Acidobacteria*)
hydrogenase maturation protease pfam01750	Ga0070308_101432	56% *Candidatus* Sulfopaludibacter sp. SbA4 (*Acidobacteria*)
hydrogenase Ni incorporation protein HypA/HybF pfam01155	Ga0070308_101433	49% *Pectinatus cerevisiiphilus* (*Firmicutes*)
hydrogenase nickel incorporation protein HypB pfam02492	Ga0070308_101434	56% *Desulfatiglans anilini* (*Deltaproteobacteria*)
hydrogenase maturation protein HypF (pfam00708, pfam01300, pfam07503)	Ga0070308_101435	66% *Chroococcidiopsis thermalis* (*Cyanobacteria*)
hydrogenase maturation protein HypC pfam01455	Ga0070308_101437	72% *Gloeothece citriformis* (*Cyanobacteria*)
hydrogenase maturation protein HypD pfam01924	Ga0070308_101438	78% *Phormidium ambiguum* (*Cyanobacteria*)
hydrogenase maturation protein, carbamoyl dehydratase HypE (pfam00586, pfam02769)	Ga0070308_101439	75% *Phormidium ambiguum* (*Cyanobacteria*)
NADP-reducing hydrogenase subunit HndB EC:1.12.1.3	Ga0070308_101418	50% *Desulfomonile tiedjei* (*Deltaproteobacteria*)
NAD(P)-dependent iron-only hydrogenase diaphorase component flavoprotein HndC, EC:1.12.1.3	Ga0070308_101419	62% *Mesoaciditoga lauensis* (*Thermotogales*)

Supplementary Table S6. A list of potential auxotrophies (as indicated by "not detected") in all three species of GAL08 at Dewar Creek.

Amino Acid	Prototrophic?
arginine	not detected
asparagine	not detected
aspartic acid	not detected
leucine	not detected
lysine	not detected
phenylalanine	not detected
serine	not detected
threonine	not detected
tryptophan	not detected
tyrosine	not detected
alanine	yes
glutamic acid	yes
histidine	yes
isoleucine	yes
proline	yes
valine	yes
cysteine	yes
glutamine	yes
methionine	yes
glycine	yes
selenocysteine	yes

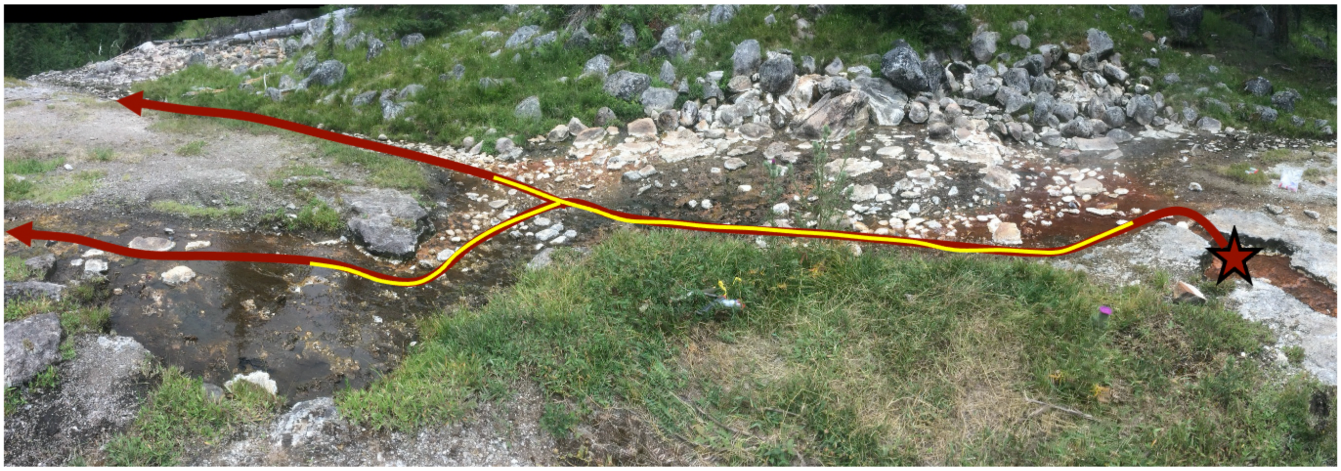

Supplementary Figure S1
Photograph of the Dewar Creek hot spring, British Columbia, as seen in July 2017. One of the outflow channel paths of the stream, which splits downstream, is marked by a red line. The source of the stream is marked by a star and the direction of the flow is indicated by arrowheads. The temperature at the source is typically around 79.8 °C; temperature gradually decreases along the flow path until ambient temperature is reached downstream, over a distance of approximately 6 meters. Areas of the outflow path in which pockets of 65°C soil can be located, and which were sampled for soils used in GAL08 cultivation experiments, are marked in yellow.

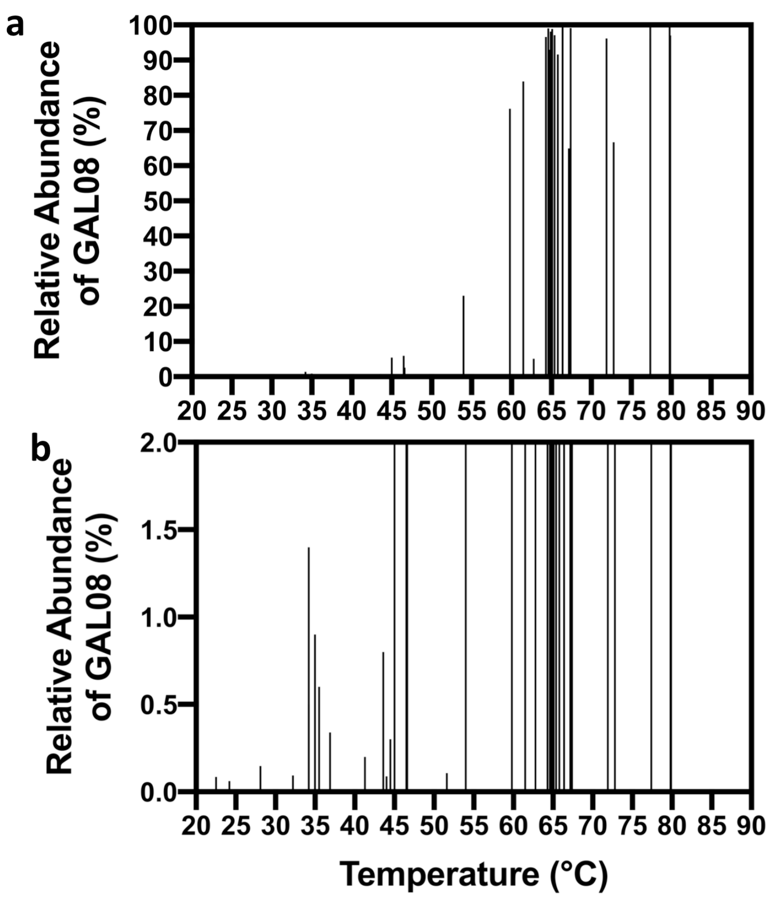

Supplementary Figure S2

Relative abundance of GAL08 relative to all *Acidobacteria* based on 16S rRNA gene amplicon sequencing of 60 bacterial communities in sediments collected from 22.5 to 85.9 °C. GAL08 reads were detected in 43 out of 60 samples. Panel (**B**) shows the same data as panel (**A**), but the y-axis is reduced to better visualize low-abundance samples.

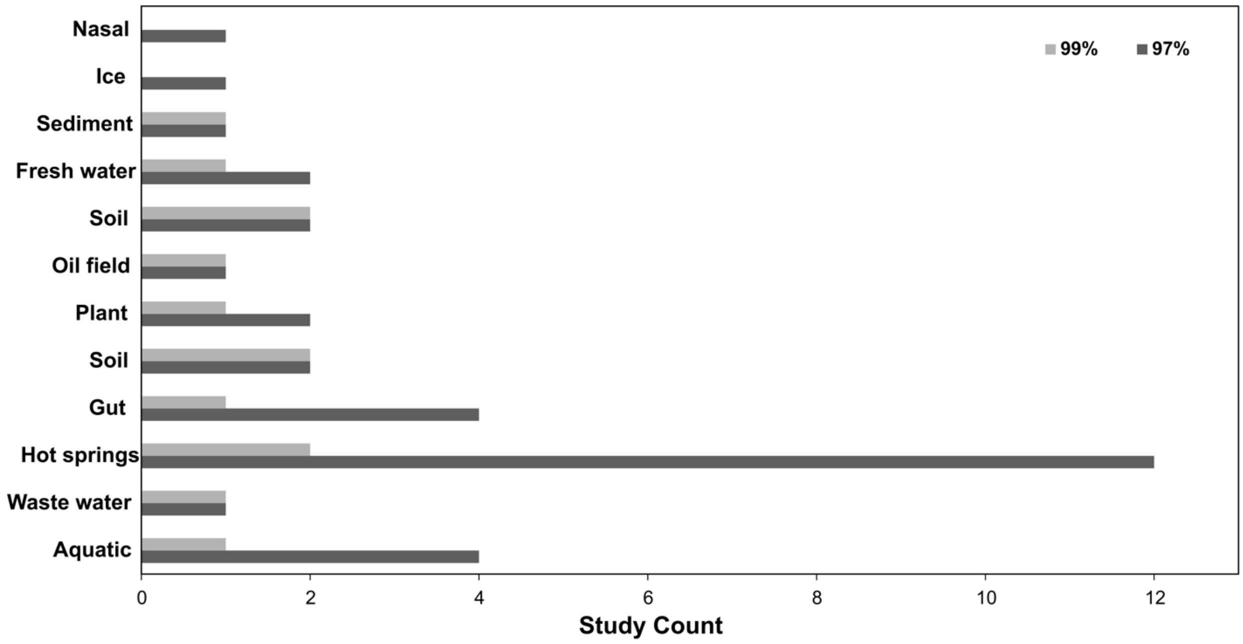

Supplementary Figure S3

Sample sites in the SRA database in which 16S rRNA gene sequences that are 97% (dark gray bars) or 99% (light gray bars) similar to DChs_GAL08 (Species 1) were detected. Data are based on a search in August 2021 using IMNGS (Lagkouvardos et al., 2016).

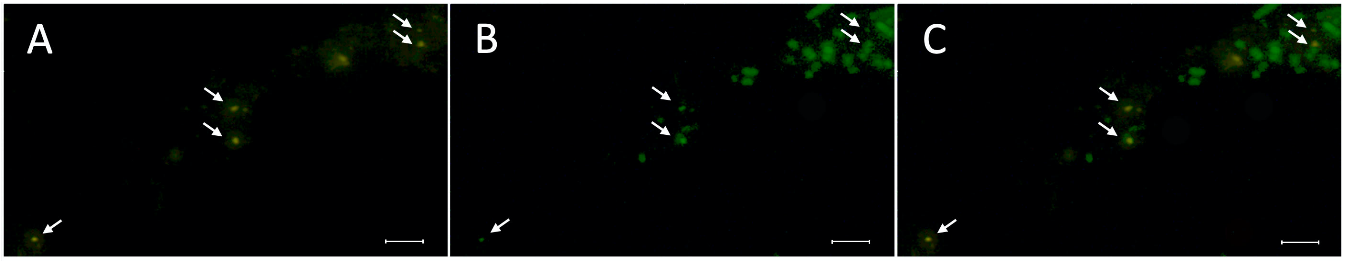

Supplementary Figure S4
Fluorescence in situ hybridization images of cells identified as GAL08 (indicated by arrows) detected by a probe mixture of GAL-08_185, cy3-labelled, yellow (**A**), EUB338, Alexa-labelled, green (**B**) in samples from Hoodoo Creek at 61 °C. A merged image of panels A and B is presented in panel (**C**). The scale bar represents 5 µm.

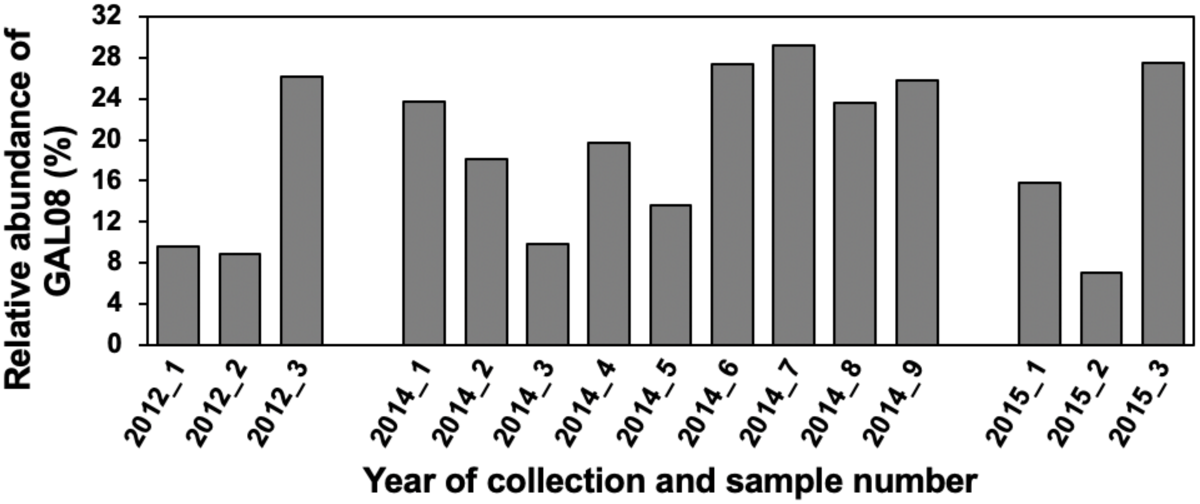

Supplementary Figure S5

Abundance of GAL08 relative to all other *Bacteria* detected at the Dewar Creek hot spring in samples collected between 64.3 and 67.4°C over three years, based on 16S rRNA gene amplicon sequencing.

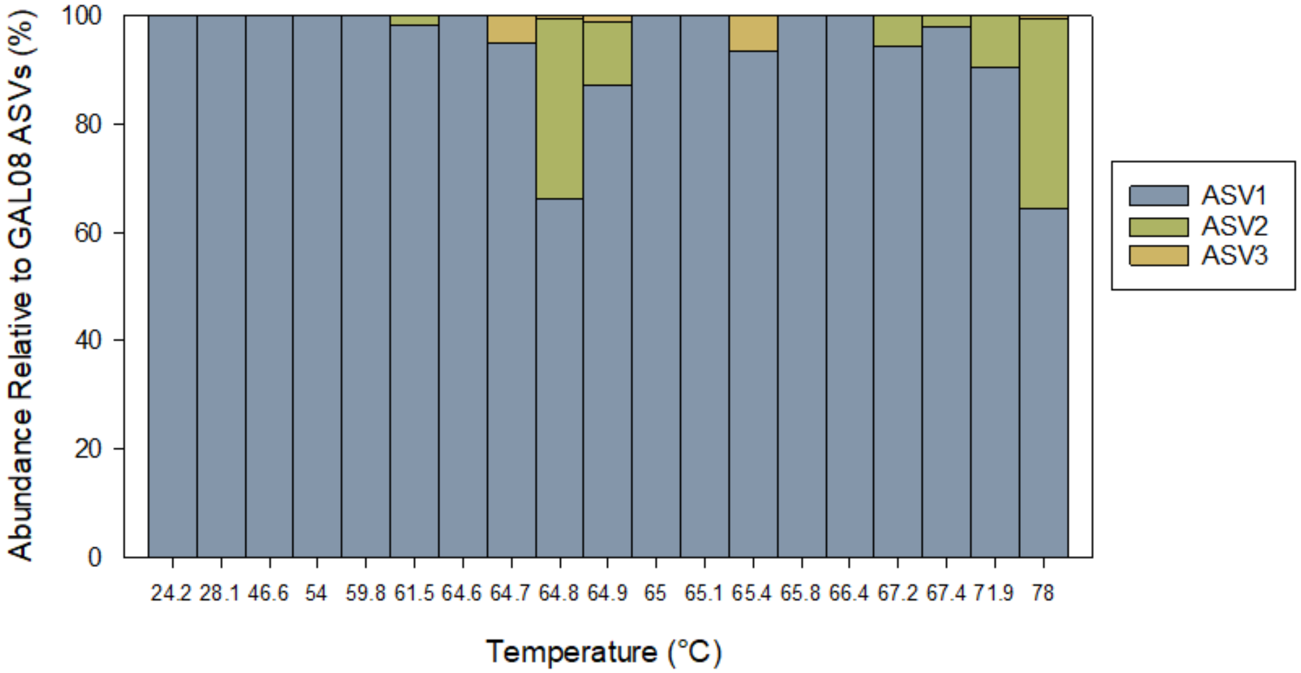

Supplementary Figure S6
Predominance of the three ASVs belonging to DChs_GAL08 at temperatures ranging from 24.1 to 78.0°C, expressed as a percentage of the entire group. ASV1=Species1, ASV2=Species2, and ASV3=Species3 (Table 1).

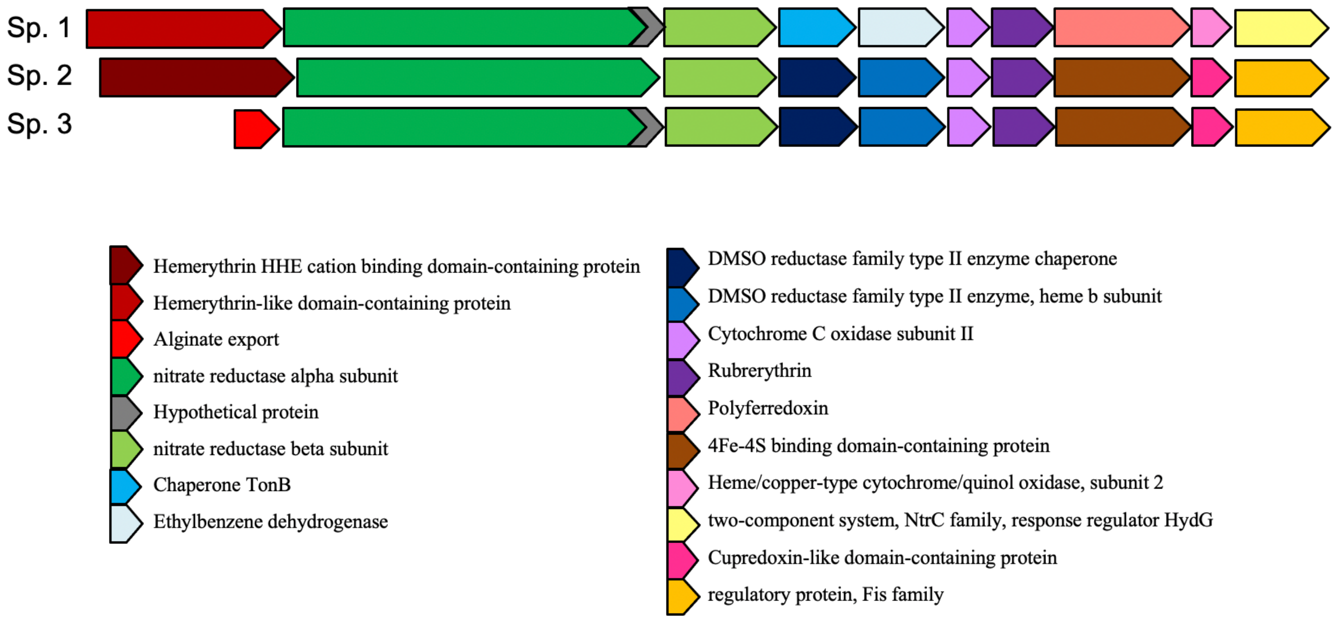

Supplementary Figure S7

Genetic environment of hemerythrin-like proteins detected in the most complete DChs_GAL08 SAGs of each species. SAGs were annotated using the JGI's IMG platform.

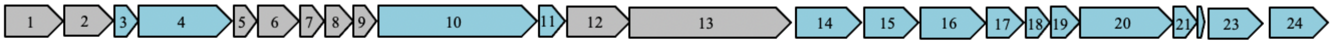

1	putative adenylylsulfate reductase-associated electron transfer protein QmoC
2	NADH:ubiquinone oxidoreductase subunit (chain E)
3	NADP-reducing hydrogenase subunit HndB
4	NAD(P)-dependent iron-only hydrogenase diaphorase component flavoprotein HndC
5	hypothetical protein
6	CBS domain-containing protein
7	DRTGG domain-containing protein
8	hypothetical protein
9	Histidine kinase-, DNA gyrase B-, and HSP90-like ATPase
10	heterodisulfide reductase subunit A
11	F420-non-reducing hydrogenase iron-sulfur subunit D
12	phenylacetyl-CoA:acceptor oxidoreductase PadC subunit
13	Anaerobic selenocysteine-containing dehydrogenase
14	cytochrome c554 and c-prime
15	F420-non-reducing hydrogenase small subunit VhoG
16	F420-non-reducing hydrogenase large subunit VhoA
17	hydrogenase maturation protease
18	hydrogenase Ni incorporation protein HypA/HybF
19	hydrogenase nickel incorporation protein HypB
20	hydrogenase maturation protein HypF
21	hydrogenase maturation protein HypC
22	hydrogenase maturation protein HypD
23	hydrogenase maturation protein

Supplementary Figure S8

Hydrogenase island in Species 2. Genes annotated as hydrogenases are colored blue, other genes are shown in gray.

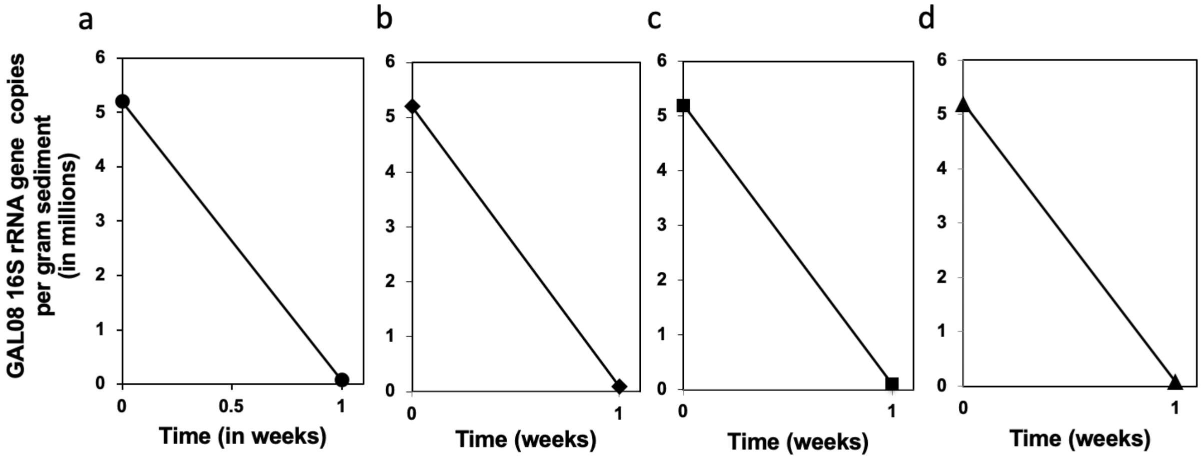

Supplementary Figure S9
GAL08 16S rRNA gene copies, in millions, per gram of sediment incubated under atmospheric conditions. The enrichment cultures used sediment and source water from the Dewar Creek hot spring, incubated at 65°C. Data shown represent unamended sediment (**A**), and sediment amended with (**B**) sodium benzoate, (**C**) trehalose, or (**D**) cellobiose.

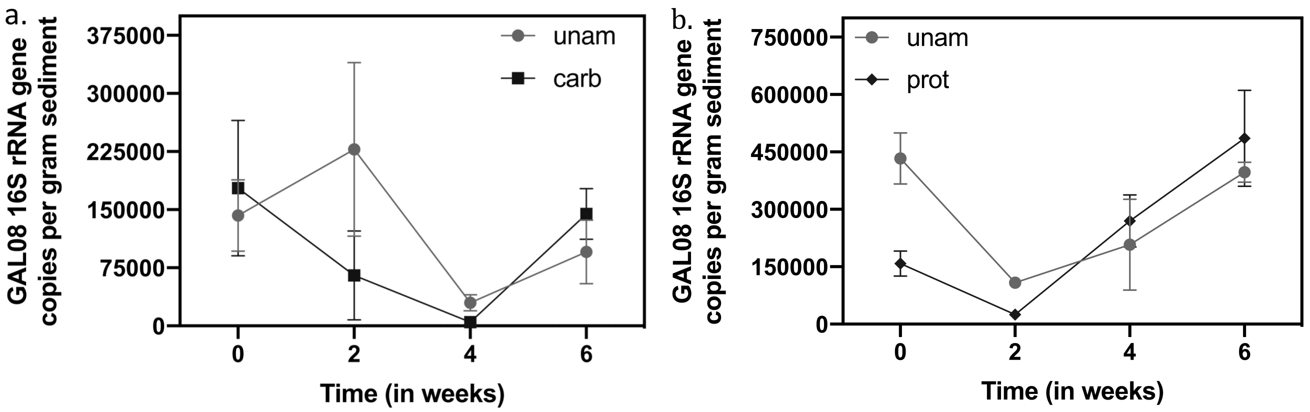

Supplementary Figure S10

GAL08 16S rRNA gene copies per gram of sediment incubated under a 5 % carbon dioxide, and 1 % oxygen atmosphere, with the remainder being nitrogen gas. All cultures used sediment and source water from the Dewar Creek both spring, incubated at 65°C, and amended with either (**A**) a carbohydrate mix (see Table S2), or (**B**) a protein mix (protease peptone, protein hydrolysate amicase, and yeast extract, at a final concentration of 0.1 g/L each, or 0.3 g/L total in the enrichment vials). Inoculating sediment was tested for the presence of GAL08 via GAL08-specific qPCR primers prior to use in the experiment. Circles, squares, and diamonds represent the mean of three replicates; error bars represent plus/minus SEM.
